# Comparative structural analysis of *Bru1* region homeologs in *Saccharum spontaneum* and *S. officinarum*

**DOI:** 10.1186/s12864-016-2817-9

**Published:** 2016-06-10

**Authors:** Jisen Zhang, Anupma Sharma, Qingyi Yu, Jianping Wang, Leiting Li, Lin Zhu, Xingtan Zhang, Youqiang Chen, Ray Ming

**Affiliations:** FAFU and UIUC-SIB Joint Center for Genomics and Biotechnology, Fujian Provincial Key Laboratory of Haixia Applied Plant Systems Biology, Haixia Institute of Science and Technology, Fujian Agriculture and Forestry University, Fuzhou, 350002 Fujian Province China; College of Life Sciences, Fujian Normal University, Fuzhou, 350108 China; Department of Plant Biology, University of Illinois at Urbana-Champaign, Urbana, IL 61801 USA; Texas A&M AgriLife Research, Department of Plant Pathology & Microbiology, Texas A&M University System, 17360 Coit Road, Dallas, TX 75252 USA; Department of Agronomy, University of Florida, 2033 Mowry Road, Gainesville, FL 32610 USA; College of Horticulture, Nanjing Agricultural University, 1 Weigang Road, Nanjing, 210095 China; College of Plant Science, Jilin University, Changchun, Jilin 130062 China

**Keywords:** Genome evolution, Haplotypes, Homologous genes, Polyploidy, *Saccharum*

## Abstract

**Background:**

Sugarcane is a major sugar and biofuel crop, but genomic research and molecular breeding have lagged behind other major crops due to the complexity of auto-allopolyploid genomes. Sugarcane cultivars are frequently aneuploid with chromosome number ranging from 100 to 130, consisting of 70–80 % *S. officinarum*, 10–20 % *S. spontaneum*, and 10 % recombinants between these two specie*s*. Analysis of a genomic region in the progenitor autoploid genomes of sugarcane hybrid cultivars will reveal the nature and divergence of homologous chromosomes.

**Results:**

To investigate the origin and evolution of haplotypes in the *Bru1* genomic regions in sugarcane cultivars, we identified two BAC clones from *S. spontaneum* and four from *S. officinarum* and compared to seven haplotype sequences from sugarcane hybrid R570. The results clarified the origin of seven homologous haplotypes in R570, four haplotypes originated from *S. officinarum*, two from *S. spontaneum* and one recombinant*.*. Retrotransposon insertions and sequences variations among the homologous haplotypes sequence divergence ranged from 18.2 % to 60.5 % with an average of 33.7 %. Gene content and gene structure were relatively well conserved among the homologous haplotypes. Exon splitting occurred in haplotypes of the hybrid genome but not in its progenitor genomes. Tajima’s D analysis revealed that *S. spontaneum* hapotypes in the *Bru1* genomic regions were under strong directional selection*.* Numerous inversions, deletions, insertions and translocations were found between haplotypes within each genome.

**Conclusions:**

This is the first comparison among haplotypes of a modern sugarcane hybrid and its two progenitors. Tajima’s D results emphasized the crucial role of this fungal disease resistance gene for enhancing the fitness of this species and indicating that the brown rust resistance gene in R570 is from *S. spontaneum*. Species-specific InDel, sequences similarity and phylogenetic analysis of homologous genes can be used for identifying the origin of *S. spontaneum* and *S. officinarum* haplotype in *Saccharum* hybrids. Comparison of exon splitting among the homologous haplotypes suggested that the genome rearrangements in *Saccharum* hybrids after hybridization. The combined minimum difference at 19.5 % among homologous chromosomes in *S. officinarum* would be sufficient for proper genome assembly of this autopolyploid genome. Retrotransposon insertions and sequences variations among the homologous haplotypes sequence divergence may allow sequencing and assembling the autopolyploid *Saccharum* genomes and the auto-allopolyploid hybrid genomes using whole genome shotgun sequencing.

**Electronic supplementary material:**

The online version of this article (doi:10.1186/s12864-016-2817-9) contains supplementary material, which is available to authorized users.

## Background

Sugarcane (*Saccharum spp.)* is an important economic crop not only owing to its contribution of approximately 75 % of world’s sugar production, but also because of its leading role in biofuel production. Modern sugarcane cultivars are mostly interspecific hybrids derived from crosses between *S. officinarum* (2n = 8x = 80) and *S. spontaneum* (2n = 40–128). Sugarcane cultivars are frequently aneuploid with chromosome number ranging from 100 to 130, consisting of 70–80 %  *S. officinarum*, 10–20 %  *S. spontaneum*, and 10 % recombinants between these two specie*s* [1], though the classical cytogenetic studies concluded that there were no chromosomal exchanges between *S. officinarum* and *S. spontaneum* after hybridization [2–4]. Molecular mapping of hybrid R570 further confirmed that interspecific chromosome exchanges occurred between the two progenitor genomes [5, 6]. GISH analyses of sugarcane hybrids indicated that the proportion of complete *S. spontaneum* chromosomes ranged from 10 % to 23 %, and recombinant chromosomes of the two parental species varied from 5 % to 17 % in the hybrids [7, 8]. Typically, the F_1_ hybrids and BC_1_ progeny receive 2n gametes from female *S. officinarum* parent and n gametes from male *S. spontaneum* parent during the interspecific hybridization, a phenomenon known as female restitution (2n + n chromosome transmission) [9].

The genome of modern sugarcane cultivar represents one of the most complex genomes studied to date. The ploidy level and genome size can vary significantly among commercial cultivars and other related *Saccharum* species. For example, the genome size of hybrid R570 was estimated at approximately 10 Gb with a ploidy level of 12x [10, 11], while the genome size of *S. officinarum* ranged from 7.50 to 8.55 Gb and that of *S. spontaneum* varied between 3.36 to 12.64 Gb [12]. So far, no sugarcane reference genome has been generated yet due to the complexity of autopolyploid genomes.

Sugarcane brown rust, caused by *Puccinia melanocephala* H&P Syd., has been a severe fungal disease impacting sugarcane production for many years. The genetic resistance of the brown rust was identified in sugarcane cultivar, R570, which was controlled by a single dose dominant gene, *Bru1*. The resistance gene was initially mapped at a location, 10 cM away from a restricted fragment length polymorphism (RFLP) probe, CDSR29 [13]. This resistance gene provides wide resistance against diverse brown rust isolates collected in both Africa and America [14]. Fine-mapping and physical mapping have been used to map this major durable resistance gene in an interval of two flanking markers, which were only 0.28 cM and 0.14 cM away from the *Bru1* gene, respectively [15–17]. A physical map covering the two flanking markers was constructed through screening the bacterial artificial chromosome (BAC) clones in the existing R570 BAC library [10] and a *Bru1*-enriched BAC library [17]. The resulting physical map included three BAC clones from the target genotype with two remaining gaps and 32 BAC clones from homologous haplotypes. Sequencing the clones in the target region revealed an inserted segment containing the target gene *Bru1* in the target haplotype contig with two gaps, and the whole segment was absent in homologous haplotype contigs [17]. Sequencing eight BAC clones, including two clones from the target haplotype and six other clones from homologous haplotypes, has revealed 14 annotated genes. The comparison of the eight BAC clones’ gapless sequences showed an average sequence identity of 97.7 % in the exons and 96.9 % in introns among the haplotypes. Based on the phylogenetic analysis of selected genes and sequence similarity of the seven haplotypes, four haplotypes were predicted to be derived from *S. officinarum,* two from *S. spontaneum*, and one being recombinant [18].

Comparative analysis between a sugarcane hybrid and its progenitor species is an effective approach to study the origin of sugarcane hybrid haplotypes, which will provide insights into chromosomal rearrangements after polyploidization and hybridization. LA Purple (*S. officinarum*, 2n = 80) and SES208 (*S. spontaneum*, 2n = 64) are varieties of the progenitor *Saccharum* species of modern sugarcane cultivars. With the available BAC libraries of LA Purple and AP85-441 (Ming and Yu, unpublished data), we investigated the sequence divergence among sugarcane hybrid (R570), *S. officinarum* (LA Purple) and *S. spontaneum* (AP85-441) in the *Bru1* genomic region. The objectives of this project were to: 1) clarify the origin of sugarcane hybrid haplotypes in the *Bru1* genomic region; 2) assess the evolutionary relationships of haplotypes within and between *Saccharum* species and interspecific hybrids; 3) evaluate the extent of DNA sequence divergence within major *Saccharum* species based on sugarcane haplotype sequences; and 4) evaluate selective constraint in genomic region containing candidate *Bru1* gene. The comparative genomic study will improve our understanding of genome recombination and evolutionary relationships of *Saccharum* hybrids and its progenitor *Saccharum* species after hybridization.

## Methods

### BAC libraries

LA Purple (*S. officinarum*, 2n = 80) and AP85-441(the haploid clone of SES208, 2n = 4x = 32) derived from the anther culture of SES208 [19] representing two major *Saccharum* species were used for BAC library construction. Nuclei were isolated from the young leaf tissues of LA Purple and SES208 haploid following the method described by Ming et al. [20]*.* The high molecular weight DNA embedded in agarose was partially digested using *Hin*dIII. The fraction at approximately 100 kb was recovered and cloned into pSMART BAC vector (Lucigen, LA). The BAC library of LA Purple consists of 74,880 clones in 195 384-well plates with average insert size at 150 kb, providing 1.5x coverage of the octoploid genome and 12x coverage of the monoploid genome. The BAC library of AP85-441consists of 38,400 clones in 100 384-well plates with average insert size at 120 kb, providing 1.5x coverage of the haploid (tetraploid) genome and 6 x coverage of the monoploid genome.

### BAC clone screening and sequencing

BAC library screening was carried out as described by Yu et al. [21]. For library screening, two probes about 500 bp were designed respectively based on the DNA sequence of two genes identified in *Bru1* region: gene 8 and 11b [18]. Among the annotated genes in the haplotypes of hybrid R570, gene 10 is homologous to barley rust resistance protein with three missing exons. Gene 8 and gene 11b, which surround gene 10, are highly conserved among the published *Saccharum* hybrid haplotype sequences [18]. Two probes corresponding to gene 8 and 11b respectively were used to screen the BAC libraries of LA Purple and AP85-441 haploid genomes (primers showed in Additional file 1).

To identify different haplotypes, the positive clones screened from the BAC library were then amplified used same primers for probes preparation, cloned into the pGEM®-T Easy Vector Systems (Promega, A1360) and sequenced from both ends of the PCR product. The BAC clones representing different haplotypes were selected. The insert sizes of the identified BAC clones were estimated by comparing to standard size markers (NEB, N3552S) using CHEF gel electrophoresis.

The BAC DNAs were isolated using phaseprepTM BAC DNA kit (Sigma-Aldrich, NA0100-1KT) and the sequencing libraries were prepared individually with unique barcode for each clone. The sequencing libraries were then pooled and sequenced using Roche 454 Genome Sequencer FLX platform at Keck Center at UIUC. The raw reads were assembled using Roche/454 Newbler Assembler with default settings (http://www.my454.com/).

### Repeat database compiling and repeat masking

To mask the repeats from the sugarcane BAC clone sequences for annotation, an in-house repeat database was compiled by assembling public available repeat databases and *de novo* assembling of sugarcane repetitive sequences.

To assemble the publicly available repeat databases, we downloaded TIGR plant repeat database (ftp://ftp.plantbiology.msu.edu/pub/data/TIGR_Plant_Repeats/) [22], the MIPS Repeat Element Database (mips-REdat) (ftp://ftpmips.helmholtz-muenchen.de/plants/REdat/) [23], Repbase (http://www.girinst.org/) [24] and the P-MITE database (without TSD for monocots from http://pmite.hzau.edu.cn/download/) [25]. Moreover, we also parsed 3470 GenBank sugarcane sequence accessions for features annotated as ‘mobile_element’, ‘LTR’, or ‘repeat_region’. The unique repeats were extracted from each downloaded repeat database by removing the redundant repeats that have more than 95 % sequence identity over 95 % of the sequence length to other repeats. Some unique repeats were annotated in RepeatMasker format (id#class/subclass) based on the repeat codes in their headers, if available, otherwise by comparing to the Repbase repeats using the RepeatClassifier script of the RepeatModeler package.

To *de-novo* identify the sugarcane repeats, available sugarcane sequences were parsed, including the sequences of the 96 sugarcane BAC clones (66 in-house BAC clones from LA Purple and AP85-441 libraries and 38 publicly available BAC clones from sugarcane hybrids (GenBank accessions AM403006-7, FJ348715-33, GU080318-23, GU207345-46, FN431661, FN431663-69, and HQ116788). The protein sequences of *A. thaliana* [26], *B. distachyon* [27], *O. sativa* [28], *S. italica* [29], *Z. mays* [30], and *S. bicolor* [31] were downloaded from Phytozome (http://www.phytozome.net/) and combined to generate an in-house plant protein database. An in-house TE protein library was also compiled from TE protein libraries available with Maker [32], GypsyDB-2.0 [33], Transposon PSI (http://transposonpsi.sourceforge.net/), and RepeatMasker [34] softwares. The *de novo* repeats were then predicted in the sugarcane BACs using the TEdenovo pipeline consisting of REPET package v2.2 [35] and using RepeatModeler—1.0.7 [36]. Gene fragments in the *de-novo* predicted repeats were identified based on their sequence similarity to plant proteins only but not TE proteins (E-value less than 0.1 using blastx) and were then N-masked. The masked repeat sequences were split on Ns and resulting sequences classified using RepeatClassifier script of the RepeatModeler package based on similarity to known repeat proteins from TREP and RepeatMasker databases. Unclassified repeats were considered as repeats if these had more than 40 matches to the 104 sugarcane BACs at E-value less than 1E-20 using blastn. Finally, the repeat database was made non-redundant using cd-hit [37, 38] with 95 % identity and 95 % coverage threshold. The final non-redundant de-novo sugarcane repeat database contained 845 repeats (representing 2, 605,348 nt) classified into 8 groups including 614 LTR retrotransposons, 167 transposons, 36 LINEs, 12 Helitrons, 8 Unknown, 6 SINEs, 1 simple repeat, and 1 satellite repeat. Sugarcane MITEs were predicted using MITEhunter [39] with default parameters.

The final in-house repeat database was then compiled by combining the unified and annotated public repeat database and the *de novo* identified sugarcane repeats. The repeat content of sugarcane BAC clone sequences in this study was determined by masking the BAC clone sequences using RepeatMasker against this compiled in-house repeat database.

### Identification of transposable elements (TE) domains and estimation of TE insertion times

To identify TE associated domains in the BAC clone sequences, rpsBLAST was used to search the BACs clone sequences against the conserved domain database (CDD) [40]. Overlapping TE domains aligned in the same orientation on the BAC clone sequences were fused as one TE domain and annotated based on the best domain hit in the CDD database. The LTR retrotransposons were identified in the sugarcane BACs based on the presence of TE domains. The two ends 5′ and 3′ LTRs were defined based on the sequence identity and the presence of target site duplications (TSD). The insertion time of full length LTR retrotransposons was calculated using the approach as described by San Mignel et al. [41]. The full length LTRs were aligned by MUSCLE [42] and the number of nucleotide substitutions per site (*k*) between the 5′ and 3′ ends of LTRs was calculated using the Kimura 2-parameter model implemented in MEGA6 [43]. The *k* values were converted to divergence time using the rate of 1.3E-8 [44].

### Gene annotation

The repeat-masked sequences were aligned against sugarcane expressed sequence tags (ESTs) comprised of 283,332 ESTs from GenBank, the unigene set of our in-house sugarcane RNAseq data, and the sorghum gene models (Sorbi1_GeneModels_AllModels_20080319_nt.fasta at http://genome.jgi-psf.org/Sorbi1/Sorbi1.download.ftp.html) using tblastx. The gene structures were further predicted using the online tool GeneSeqer (http://www.plantgdb.org/cgi-bin/GeneSeqer/index.cgi), and the ambiguities were checked and manually corrected according to the alignment of the sequences to sugarcane transcripts and sorghum gene models. The annotated genes from the sequences were compared to 52 corresponding genes in seven haplotypes from hybrid R570 (GenBank accessions: FN431661-FN431668) and 10 corresponding genes from a sorghum BAC (GenBank accessions: FN431669) [18].

To estimate the expression level of annotated genes in different tissues of LA Purple and AP85-441, we aligned RNAseq data of 42 million pair-end reads 20 million single-end reads from various tissues of LA Purple and AP85-441 respectively against the predicted cDNA sequences of annotated genes using Novoalign with default settings (http://www.novocraft.com/main/index.php). The number of aligned reads for each target gene were counted using Tablet [45]. The gene expression levels were calculated as fragments per kilobase of exon per million mapped fragments (RPKM) [46].

### Sequence divergence analysis

Protein sequences of gene pairs were aligned with ClustalW 2.0 [47] The alignments were converted to codon alignment with PAL2NAL [48]. The substitution rates of synonymous (Ks) and non-synonymous (Ka) were estimated based on the YN method [49] using KaKs_Calculator 2.0 [50].

Ka/Ks value differential significance analyses were performed using fisher exact test as implemented in KaKs_Calculator 2.0 [49]. The null hypotheses in fisher exact test is numbers of synonymous substitutions (Sd)/number of synonymous sites (S) = number of nonsysnonymous substitutions (Nd)/number of nonsysnonymous sites (N), also means neutral mutation. Reject the null hypothesis if Sd/S is significantly greater (negative selection) or smaller (positive selection) than Nd/N, as indicated by P-value < 0.05, and extremely significant if *P*-value < 0.01. On the other hand, significance analyses for different groups of Ka/Ks values was performed using Duncan’s test with significance level of 0.05, which was implemented in agricolae package of R programming language [51]. A custom Perl script was used for SNP discovery based on pairwise sequence alignments (https://github.com/lileiting/Pileup2singledose/tree/master/dnp).

Furthermore, we applied DnaSP 4.0 [49] to perform sliding window analysis for nucleotide diversity, Tajima’s D and Fu and Li’s D test with 1 kbp window size and 100 bp step length.

### BAC sequence visualization and comparison

The schematic of exons, conserved TE domains and repeats in sugarcane BAC sequences was generated using EasyFig [52]. Large-scale alignments between homologous BACs were performed using BLASTZ [53]. The BAC sequence comparison was performed using the Artemis Comparison Tool [54] and a genome alignment tool Mauve with default settings (http://gel.ahabs.wisc.edu/mauve/) [55].

## Results

### Screening and sequencing *S. officinarum* and *S. spontaneum* BAC clones containing *Bru1* genomic region

Nine positive clones from LA Purple and five from AP85-441 were identified using two probes designed from genes 8 and 11 of the *Bru1* genomic region*.* Among them, five from LA Purple and three from AP85-441 were confirmed by PCR. To distinguish the haplotypes and avoid sequencing the duplicated haplotypes, PCR fragments of gene 8 were cloned and sequenced, which confirmed four clones from LA Purple (So-57E04, So-96B11, So-99P01 and So-146H19) and two clones from AP85-441 BAC libraries (Ss-75D04 and Ss-23 K06) containing different homologous haplotypes. The insert sizes of six BAC clones ranged from 80 kb to 130Kb. These six clones were subjected to complete sequencing. The cleaned reads from each clone were assembled and yielded a total length of 559 kb for the six clones with average GC contents ranging from 44.2 % to 46.8 % (Table 1). The sequences of the six clones were deposited in Genbank (accession numbers: KP063111- KP063116).

**Table 1:** Summary of the sequence length, GC content, transposable element content, and gene number

Species	NO	BAC ID	Length (Kb)	GC content	Transposable elements			Gene number
					LTR	Non-LTR	Transposons	
*S. officinarum* (LA Purple)	1	146H19	77.5	44.7 %	16.91 %	1.92 %	29.47 %	8
	2	99P01	74.4	44.2 %	35.94 %	2.46 %	28.97 %	7
	3	96B11	101.3	46.8 %	35.12 %	0.05 %	24.55 %	8
	4	57E04	95.3	45.0 %	34.90 %	3.57 %	16.42 %	8
*S. spontaneum* (AP85-441)	5	75D04	72.0	44.7 %	11.13 %	6.57 %	36.23 %	10
	6	23 K06	127.7	45.3 %	26.66 %	4.70 %	25.31 %	11
Average			91.5	45.2 %	27.48 %	3.20 %	26.16 %	8.7
Average (LA)					31.19 %	1.94 %	24.36 %	
Average (SES)					21.02 %	6.14 %	29.27 %	
Total			549	-				52

Fifty-two genes were annotated from the sequences of the six BAC clones (Table 1 and Additional file 2). The average gene density was 1 gene/11.2 kb in LA Purple and 1 gene/9.5 kb in AP85-441 homologous BAC clone sequences. The total coding regions of predicted genes account for 35 % and 38 % of the sequences from LA Purple and AP85-441, respectively.

### Sequence comparison between homologous haplotypes

Comparative analysis was performed between the homologous BAC sequences of LA Purple, So-57E04, So-96B11, So-99P01 and So-146H19. Pair-wise sequence alignments revealed insertions and deletions in all six pairs (Additional file 3: Figure S1.1–1.6 and Additional file 4). The large InDels were observed mainly in inter-genic regions between gene 5 and gene 6, gene 6 and gene 7, and within the genic regions of gene 4, gene 5, gene10 and gene 11a (Table 2). The alignment gaps appeared in all pairs and were not equally distributed, ranging from 18.2 % to 60.5 % (33.7 % in average) of the aligned regions. The four haplotypes shared identities of approximately 96.8 % in average ranged from 95.44 % to 98.39 %, and an average of 1.8 % SNPs difference with a range of 1.66 % to 2.48 % on the gapless alignments of the corresponding regions (Table 3). The average divergence among the haplotypes in *S. officinarum* is 3.2 %. Besides, inversions were clearly observed in alignments of all pairs excepting the So-57E04/So-96B11 (Additional file 3: Figure S1.6).

**Table 2:**
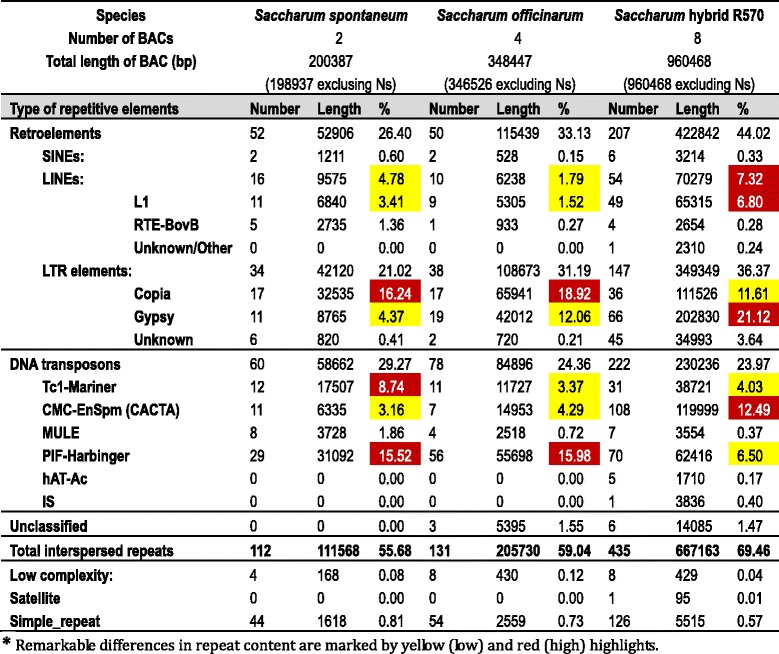
Repeat content in the haplotype sequences of LA Purple (*S.officinarum*), AP85-441 (*S. spontaneum*), and the hybrid cultivar, R570

**Table 3:** Summary of gapless sequence comparison the haplotypes BACs among *S. officinarum*(LA Purple) and *S. spontaneum*(AP85-441)

BAC name	BAC name	Ss-75D04	So-57E04	So-96B11	So-99P01	So-146H19
(Length bp)	Length (bp)	71,995	95,342	101,291	74,354	77,460
Ss-23 K06	range of aligned sequence	(287–88918)/(302–72582)	(39884–95090)/(17837–88219)	(168–81137)/(8608–80258)	(37335–85913)/(26–66382)	(6628–78055)/(5672–77310)
127,658	Span of aligned BAC	68512(77.30 %)/68716(95.07 %)	33316(60.35 %)/33467(47.55 %)	51174(63.20 %)/51331(71.64 %)	29192(60.09 %)/29187(43.99 %)	49413(69.18 %)/49502(69.10 %)
	Aligned sequence	67783	32441	50240	28799	48249
	Average identity (%)	98.37	95.92	96.08	96.72	94.53
	SNP (%)	881(1.30 %)	620(1.91 %)	1035(2.06 %)	506(1.76 %)	1153(2.39 %)
	MNP(%)	0.43	2.17	1.86	1.52	3.08
Ss-75D04	range of aligned sequence		(35029–72582)/(17837–83938)	(302–65299)/(8743–80258)	(32480–72726)/(26–74352)	(6303–62979)/(5672–77310)
71,995	Span of aligned BAC		29553(78.70 %)/29618(44.81 %)	49688(76.45 %)/49719(69.52 %)	29384(73.01 %)/29378(39.53 %)	46112(81.36 %)/46037(64.26 %)
	Aligned sequence		28814	48973	28938	45233
	Average identity (%)		95.80	96.48	97.00	96.36
	SNP (%)		479(1.66 %)	984(2.01 %)	489(1.69 %)	1123(2.48 %)
			2.54	1.51	1.31	1.16
So-57E04	range of aligned sequence			(9475–36844)/(39893–80175)	(17837–95341)/(6441–67799)	(10894–39851)/(28927–77310)
95,342	Span of aligned BAC			21851(79.84 %)/21895(54.35 %)	57223(73.83 %)/57303(93.39 %)	24491(84.58 %)/24304(50.23 %)
	Aligned sequence			21601	56769	23815
	Average identity (%)			95.99	97.28	95.44
	SNP (%)			377(1.75 %)	993(1.75 %)	473(1.99 %)
	MNP(%)			2.26	0.97	2.57
So-96B11	range of aligned sequence				(48130–80175)/(38–27603)	(14403–80175)/(5672–71165)
101,291	Span of aligned BAC				23235(72.51 %)/23221(84.24 %)	47909(72.84 %)/47707(72.84 %)
	Aligned sequence				23083	46867
	Average identity (%)				98.39	96.57
	SNP (%)				364(1.58 %)	853(1.82 %)
	MNP(%)				0.03	1.61
So-99P01	range of aligned sequence					(24–30055)/(34688–77310)
74,354	Span of aligned BAC					24568(81.81 %)/24554(57.61 %)
	Aligned sequence					24266
	Average identity (%)					96.98
	SNP (%)					441(1.82 %)
	MNP(%)					1.20

Notes: A summary of gapless sequence comparison the haplotypes BACs among *S.officinarum*(LA Purple), *S.spontaneum*(AP85-441) and *Saccarhum* cultivar R570 was presented in table S. 4. SNP: single nucleotide polymorphism; MNP: Multiple nucleotide Polymorphisms

**Fig. 1:**
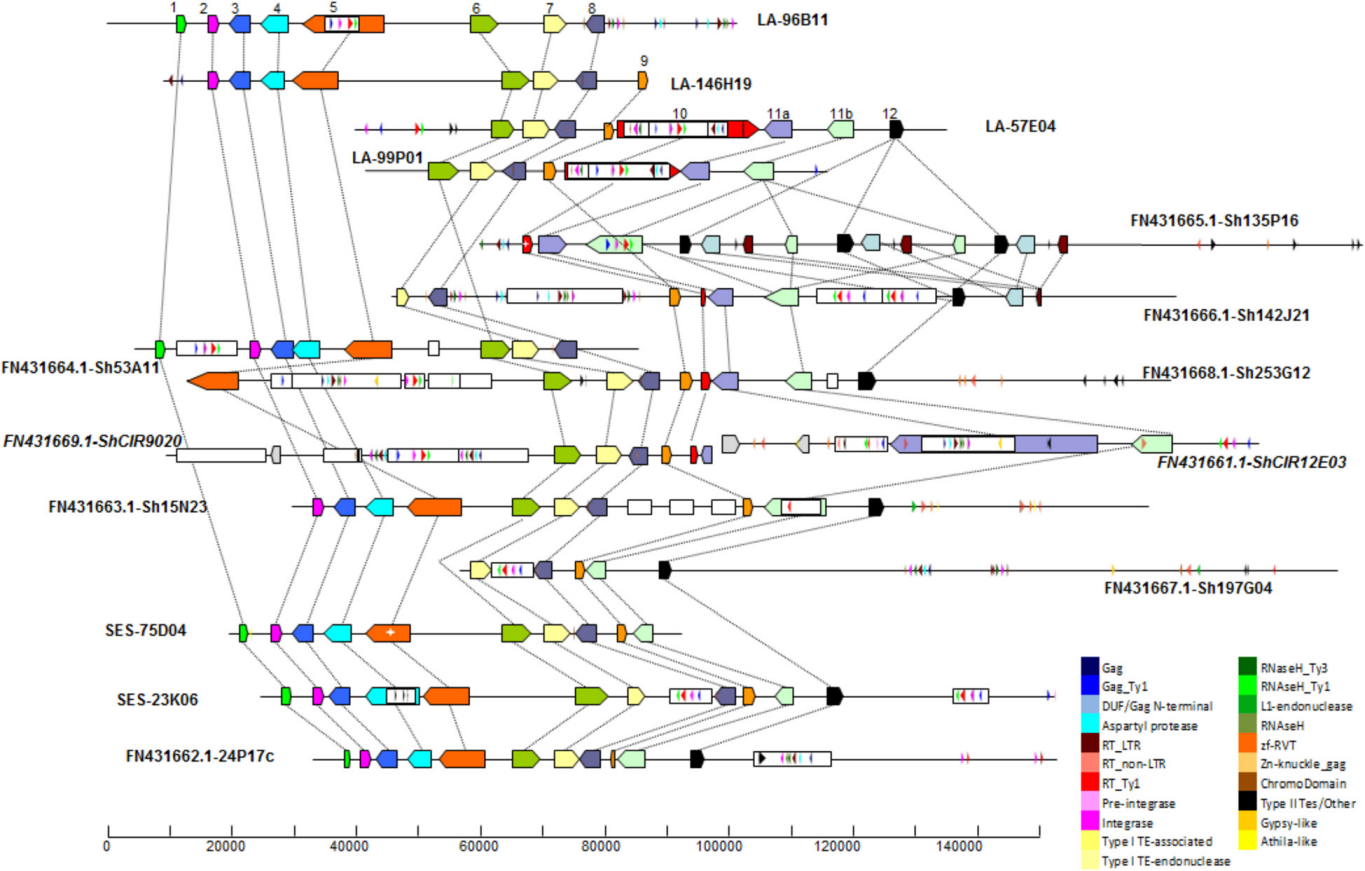
Comparison of the genome structures between 14 haplotypes (15 BAC sequences) from LA Purple (*S.officinarum*), AP85-441 (*S. spontaneum*), the hybrid cultivar R570, and sorghum. Genes are presented by color pentagon boxes. Psudogenes are marked with star, and TEs are indicated by rectangle and showed by color. Genes are numbered according to Additional file 2

Between the homologous sequences of *S. spontaneum* BAC clones, Ss-75D04 and Ss-23 K06, 72,274 bp (from 302 bp to 72,576 bp) from Ss-75D04 were aligned with 88,625 bp (from 287 bp to 88,912 bp) from Ss-23 K06, showing a 16,351 bp (18.4 %) expansion in Ss-23 K06. These two BACs shared an average sequence identity at 98.47 % and had 1.30 % of SNPs on the gapless comparison. InDels were observed between these two BACs (Additional file 3: Figure S1.7). Two large transposable elements, belonging to DNA/MULE-MuDR and LTR/Copia families, were found at regions 19,051–23,834 and 62,361–71,846 in Ss-23 k06, respectively (Fig. 1 and Additional file 5). In the genic regions, a 4,818 bp insertion in intron 2 of gene 4 and a 566 bp insertion in intron 4 of gene 5 were found on the Ss-23 K06 (Fig. 1 and Additional file 6: Figure S 4.5), presenting a 14.3 % (5,374/37,661, length of insertions/length of 11 genes) expansions in genic region of the BAC sequences. These results indicated that the expansion on haplotype of Ss-23 K06 was originated from transposable element insertions in both genic and intergenic regions.

Sequence comparison between haplotypes of *S. spontaneum* and *S. officinarum* showed large InDels in intergenic regions between two pairs of genes: genes 5 and 6, genes 6 and 7, and within the genic regions of two pairs of genes: genes 4 and 5, genes 10 and 11a. In addition, large segmental insertions were observed in intergenic regions between genes 9 and 11b in all the haplotypes of *S. officinarum*, which can be used as a *S. officinarum*-specific marker to distinguish the origin of haplotypes in hybrids. Unevenly distributed alignment gaps existed in all eight pairs of BACs ranging from 18.2 % to 60.5 % of the aligned sequence. Inversions were found in the corresponding regions between genes 5 and 6 of paired haplotypes of Ss-23 K06/So-146H19 and Ss-75D04/So-146H19. Further sequence analysis has shown that the inverted segment of 48,541–51,370 in So-146H19 might be originated from the duplication and inversion of the segment of 45,239–48,068 (Additional file 3: Figure S1.15). Based on the gapless alignments of the corresponding regions, the two haplotypes of *S. spontaneum* shared an average of 96.1 % (ranging from 94.5 % to 97.0 %) sequence identities and showed an average of 2.0 % SNPs (ranging from 1.7 % to 2.5 %) with the four haplotypes from *S. officin*arum (Table 3).

### Identification of SNPs in the *Bru1* homologous haplotypes

In the *Bru1* homologous haplotypes, pairwise sequences alignments were performed for discovering SNPs within and among *Saccharum* Species in the gapless regions (Table 4). 3501, 881 and 6389 SNPs corresponding to 196,401 bp, 67,783 bp and 311,687 bp of aligned sequences were identified within *S. officinarum*, *S. spontaneum* and between *S.officinarum* and *S. spontaneum*, respectively. The SNP densities were higher in between *S. officinarum* and *S. spontaneum* than within each of the two *Saccharum* species.

**Table 4:** Pairwise SNPs distributions in *Saccharum* species

	Aligned sequence(bp)	SNP number	SNP %	100 nt		250 nt		500 nt		1000 nt	
				SD	0 SNPs (%)	SD	0 SNPs (%)	SD	0 SNPs(%)	SD	0 SNPs(%)
So	196401	3501	1.78	1.73	31.35	3.57	11.26	6.52	2.77	11.77	0.40
Ss	67783	881	1.30	1.34	50.22	2.94	27.52	5.51	22.30	9.78	17.39
Ss/So	311687	6389	2.05	1.76	18.66	3.54	3.31	6.27	0.49	11.32	0.00

Notes

The SNPs discovery was based on pairwise gapless sequence comparisons

0 SNPs (%): The percentage of fragments for specific sizes (100 nt, 250 nt, 500 nt and 1000 nt) that contained no SNP

SD: The standard variation of SNP numbers among the specific sizes of fragments

So: *S. officinarum*, Ss: *S. spontaneum*

To evaluate the distributions of SNPs and the possibility of distinguishing the homologous haplotypes in *Saccharum* genomes, the SNP number of the fragment with sizes of 100 bp, 250 bp, 500 bp and 1000 bp were identified in the pairwise alignment in the *Bru1* homologous haplotypes. The results revealed that 50.22 % of 100 bp sequences pairs and 27.52 % of SNPs of 250 bp sequences pairs had no SNPs in *S. spontaneum. S. spontaneum* were observed to exhibit more identical sequences between the two *Bru1* homo(eo)logous haplotypes (Table 4).

### Identification of species-specific haplotypes in R570

Segmental InDels between gene 10 and gene 11b were presented in *S. officinarum* LA Purple and absent in *S. spontaneum* AP85-441. These InDels can be used to identify the species-specific haplotypes in *Saccharum* hybrid R570**.** Based on the large *S. officinarum* insertion fragment, the six haplotypes from hybrid R570 can be sorted into two groups, one group including BACs 142 J21, 135P16, 253G12 and CIR9020/12E03 with the insertion as in *S. officinarum*, and the other group including BACs 15 N23 and 197G04 without the insertion as in *S. spontaneum*. 53A11 was not grouped together with the other BACs because it does not have the corresponding homolog sequence (Table 5 and Fig. 1).

**Table 5:** The feature of syntenic genes on *Saccharum* and sorghum bacterial artificial chromosome (BAC) clones

		*S. spontaneum*		*S. officinarum*				Saccharum hybrid-*S. spontaneum*		*Saccharum* hybrid-*S. officinarum*				*Saccharum* hybrid recombination	*Sorghum*
		Ss-75D04	Ss-23 K06	So-99P01	So-57E04	So-96B11	So-146H19	15 N23	197G04	142 J21	135P16	253G12	53A11	CIR9O20/12E03	24P17
															Sorghum
Gene1	DNA	810	813	-	-	813	-	-	-	-	-	-	996	-	561
	Exons	1	1	-	-	1	-	-	-	-	-	-	2	-	1
	cDNA	810	813	-	-	813	-	-	-	-	-	-	741	-	561
	Amino acids	269	270	-	-	270	-	-	-	-	-	-	246	-	186
Gene2	DNA	1180	1195	-	-	1195	1195	1192	-	-	-	-	1195	-	1192
	Exons	2	2	-	-	2	2	2	-	-	-	-	2	-	2
	cDNA	1086	1101	-	-	1101	1101	1098	-	-	-	-	1101	-	1098
	Amino acids	361	366	-	-	366	366	365	-	-	-	-	366	-	365
Gene3	DNA	3216	3214	-	-	3223^a^	3356	3449	-	-	-	-	3203	-	3193
	Exons	7	7	-	-	7	7	7	-	-	-	-	7	-	7
	cDNA	948	948	-	-	953^a^	948	975	-	-	-	-	948	-	975
	Amino acids	315	315	-	-	-^a^	315	324	-	-	-	-	315	-	324
Gene4	DNA	3894	8712	-	-	3910	3516	3888	-	-	-	-	3515	-	3330
	Exons	10	10	-	-	10	10	10	-	-	-	-	10	-	10
	cDNA	1002	1002	-	-	1002	993	960	-	-	-	-	1002	-	1032
	Amino acids	333	333	-	-	333	330	319	-	-	-	-	333	-	343
Gene5	DNA	6972^a^	6954^a^	-	-	12770	7312	7912	-	-	-	7328	7300	-	6964
	Exons	14^a^	14^a^	-	-	14	14	14	-	-	-	14	14	-	14
	cDNA	2706^a^	2671^a^	-	-	2667	2676	2676	-	-	-	2586	2670	-	2529
	Amino acids	-^a^	-^a^	-	-	888	891	891	-	-	-	861	889	-	842
Gene6	DNA	3986	4542	3996	3987	3999	4007	3958	-	-	-	3990	3988	4294	4144
	Exons	8	8	8	8	8	8	8	-	-	-	8	8	8	7
	cDNA	876	873	873	873	873	873	882	-	-	-	882	882	882	891
	Amino acids	291	290	290	290	290	290	293	-	-	-	293	293	293	296
Gene7	DNA	3829	3779	3782	3805	3781	3775	3977	3298#	1876#	-	3814	3812	3962	3717
	Exons	8	8	8	8	8	8	8	7#	6#	-	8	8	8	8
	cDNA	1092	1092	1092	1092	1092	1092	1092	1023#	966#	-	1092	1092	1092	1086
	Amino acids	363	363	363	363	363	363	363	340#	321#	-	363	363	363	361
Gene8	DNA	2988	2982	2974	2991	2993^a^	2991	2976	2982	2993	-	2991	2991	3003	3003
	Exons	5	5	5	5	5^a^	5	5	5	6	-	5	5	5	5
	cDNA	2412	2421	2376	2415	2417^a^	2415	2406	2421	2367	-	2415	2415	2427	2421
	Amino acids	803	806	791	804	-^a^	804	801	806	788	-	804	804	808	806
Gene9	DNA	1632	1638	1632	1626	-	-	1632	1413	1632	-	1629	-	1575	X
	Exons	1	1	1	1	-	-	1	1	1	-	1	-	1	X
	cDNA	1632	1638	1632	1626	-	-	1632	1413	1632	-	1629	-	1575	X
	Amino acids	543	545	543	541	-	-	543	470	543	-	542	-	524	X
Gene10	DNA	X	X	17423^a^	22357^a^	-	-	X	X	620^a^	744^a^	1057^a^	-	1066	X
	Exons	X	X	6^a^	6^a^	-	-	X	X	3^a^	5^a^	4^a^	-	6	X
	cDNA	X	X	490^a^	436^a^	-	-	X	X	427^a^	393^a^	501^a^	-	534	X
	Amino acids	X	X	N/A ^a^	N/A ^a^	-	-	X	X	N/A^a^	N/A ^a^	166^a^	-	177	X
Gene11a	DNA	X	X	4273	4059	-	-	X	X	4043	4060	4062	-	1566#/33480	X
	Exons	X	X	7	6	-	-	X	X	6	6	6	-	4#/6	X
	cDNA	X	X	1029	1029	-	-	X	X	1029	1029	1029	-	645#/993	X
	Amino acids	X	X	342	342	-	-	X	X	342	342	342	-	214#/330	X
Gene11b	DNA	3293	3832	3671	3678	-	-	9988	3271	5684	9146	3674	-	6477	4616
	Exons	6	6	6	6	-	-	6	6	7	6	6	-	7	6
	cDNA	948	948	948	948	-	-	948	948	927	948	948	-	954	957
	Amino acids	315	315	315	315	-	-	315	315	308	315	315	-	317	318
Gene12	DNA	-	1836	-	1841	-	-	2053	1840	1877	1877/1881/1881^1^	1841	-	-	1846
	Exons	-	3	-	3	-	-	3	3	3	3/3/3	3	-	-	3
	cDNA	-	1536	-	1539	-	-	1146	1536	1539	1671/1611/1611	1539	-	-	1545
	Amino acids	-	511	-	512	-	-	381	511	512	556/536/536	512	-	-	514

Notes: ^a^ pseudo gene # BAC border X deletion - out of BACs

1. Three genes of 12 were annotated in the BAC 135P16 of R570

The sequence comparisons provided a reference for distinguishing the haplotype origin in *Saccharum* hybrids. The four haplotypes with the large inserted fragment from R570 shared higher sequence similarity (96.62 to 98.38 %) with *S. officinarum* than that with *S. spontaneum* (94.56 to 95.88 %) (Table 5)*.* The sequence of BAC clone 53A11 missing the corresponding insertion also presented higher sequence similarity (96.41 %) with *S. officinarum* than that with *S. spontaneum* (95.67 %). BACs 15 N23 and 197G04 shared 96.07 % and 97.24 % sequences identities with *S. spontaneum* haplotypes*,* and 96.61 and 93.82 % with *S. officinarum* haplotypes, respectively (Table 6)*.*

**Table 6:** The average sequence identities between the homologous haplotypes from two progenitors and *Saccharum* hybrids R570

BAC ID	15 N23	197G04	142 J21	135P16	253G12	53A11	CIR9020/12E03
Haplotype ID	I	III	V	VI	II	IV	VII
Genbank ID	FN431663.1	FN431667.1	FN431666.1	FN431665.1	FN431668.1	FN431664.1	FN431669.1/FN431661
Length (bp)	137851	141630	126547	142236	158483	81164	87631 + 84926
*S. spontaneum* haplotypes	96.07	97.24	95.88	94.56	95.28	95.87	95.67
*S. officinarum* haplotypes	96.61	93.82	98.38	97.48	96.65	96.62	96.41

**Fig. 2:**
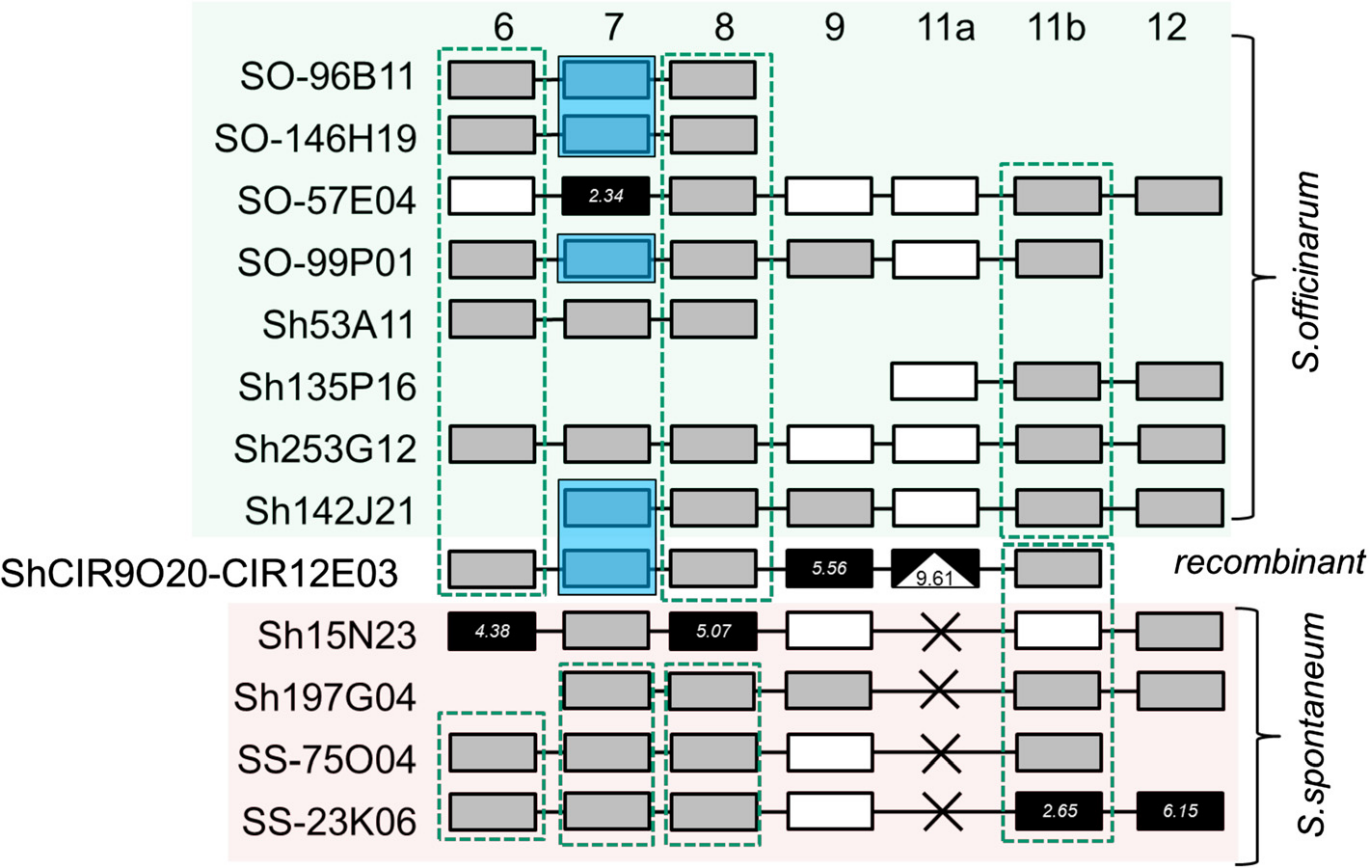
Schematic representation of verifying the homologous haplotype origin in *Saccharum* hybrid R570 based on homo(oe)ologous gene allele sequence comparison with two progenitor *Saccharum* species as references. Notes: Similar analyses as Garsmeur et al. (2011) [18] were performed for the schematic. Each gene allele is represented by a square. For each locus, the most divergent allele is marked in black and its theoretical divergence time (highest estimate observed in Myr) is indicated in italics. All alleles that fall into groups (of at least three) with all values lower than one-third of this maximum divergence time are marked by white squares. When the phylogenetic trees were not degenerate, the alleles (loci 6, 7, 8, and 11b) of the same branch (relating to the same internal node) were placed in vertical dotted boxes. But for five alleles of loci 7, the alleles from the same phylogenetic tree branch were placed in the transparent blue boxes due to they distribute separately in the figure. The white triangle in the black square for locus 11a indicates an insertion. The ‘x’ mark indicates absence of the gene

Furthermore, to verify the prediction by Garsmeur et al. [18], similar analyses with phylogenetic tree and haplotype networks were used to identify the origin of the *Bru1* region in *Saccharum* hybrids with homologous sequences from two progenitor *Saccharum* Species as references. To be comparable, a similar synthetic representation as Garsmeur et al. [18] for the results is presented in Fig. 2. The maximum divergence between two alleles within a locus ranges from 2.34 to 9.61 MYRs. Sh15N23, CIR9020-12E03 and Ss-23 K06 contain two of the most divergent gene alleles. Based on the phylogenetic analysis of gene alleles, genes 6, 8, and 11b were all grouped separately from *S. spontaneum* and *S. officinarum* haplotypes; gene 7 from two *S. spontaneum* and three out of four *S.officinarum* haplotypes (beside So57E04) were grouped. Therefore, the hybrid BACs 142 J21, 135P16, 253G12, and 53A11 should be originated from *S. officinarum* as shown by phylogenetic groups of genes 6, 7 and 8*,* while, hybrid BACs 15 N23 and 197G04 should be from *S. spontaneum* according to phylogenetic group of genes 6, 7, 8 and 11b (Fig. 2). In addition, in haplotype CIR9020-12E03, genes 6, 7, and 8 were grouped with the *S. officinarum* alleles, and gene 11a in the *S. officinarum* specific InDel region was presented, while, 11b was grouped together with the *S. spontaneum* alleles. This result demonstrated that CIR9020-12E03 was a haplotype with a recombinant region between gene 11a and 11b. Our results confirmed the prediction by Garsmeur et al. that of the seven haplotypes from *Saccharum* hybrids, four were derived from *S. officinarum,* two from *S. spontaneum* and the remaining one was from recombinant.

### Gene arrangements and structures in homologous haplotypes

The lack of genes 10 and 11a was observed in haplotypes of *S. spontaneum* (AP85-441) in comparison to haplotypes of *S. officinarum* (LA Purple) (Fig. 1 and Table 5). Similarly,, genes 10 and 11a were only found in our in-house RNA-seq database of *S. officinarum*, but not in *S. spontaneum*. Except these two genes, all the other genes remained the same order and orientation with conserved sizes and coding sequences in both species. Pseudogenes were found in alleles of both *Saccharum* species. In the two haplotypes from *S. spontaneum* (AP85-441), a premature stop codon caused by an insertion was found in the exon 11 of gene 5. In *S. officinarum*, a premature stop codon was also observed in the exon 6 of gene 3 and exon 1 of gene 8 in the haplotype of So-96B11, which resulted two pseudogenes. Two large insertions, 21 kb and 17 kb, were found in the introns of genes 10 in haplotypes of So-57E04 and So-99P01, respectively (Additional file 6). 4 out of the 28 genes (gene 10 was not included) in two *Saccharum* species were identified to be pseudogenes (Table 5).

Comparing the genomic region in two species and hybrid R570, the genes in haplotypes of 15 N23 and 197G04 from R570 remained the same order, orientation and missed genes (10 and 11a) as presented in the haplotypes of *S. spontaneum* (AP85-441). The remaining five of six haplotypes from hybrid BACs showed the same order and orientation as in haplotypes of *S. officinarum* (LA Purple). All the sequence alignment and gene comparison indicated that the two haplotypes of 15 N23 and 197G04 are originated from *S. spontaneum*, validating prediction of Garsmeur et al. [18]. In hybrids, except gene 10, the coding regions of all the other genes could be translated into complete protein sequences [18].

Comparing homologous sequences between sugarcane and sorghum, gene 9, gene 10 and gene 11a were absent in sorghum. Only the first exon of gene 9 was retained in sorghum. Large InDels were found in the region between genes 8 and gene 11b between sugarcane and sorghum, which might indicate that the region between genes 8 and gene 11b was a hotspot of genome rearrangement in *Saccharum*.

The structure of each gene was analyzed. The gene size differences were mainly caused by the variations of intron length. Except for genes 1, 2 and 9, the other 10 unique genes exhibited distinct sizes of introns among the haploytypes (Additional file 6). Furthermore, LTR insertions were found in genes 4, 10, 11a and 11b, which caused the intron size variation in these genes. However, the intron variations occurred randomly among different haplotypes. By contrast, coding regions were conserved among haplotypes. Of the 13 unique genes, exon splitting occurred in genes 1 and 8 of haplotype ShIV (FN31664.1) and ShV (FN31666.1) from the hybrid, respectively. However, these genes preserved their coding and putative amino acid sequences among all the alleles in the two progenitor species.

### Selective constraints on homologous genes between the haplotypes of *S. officinarum, S. spontaneum* and *Saccharum hybrid*

**Fig. 3:**
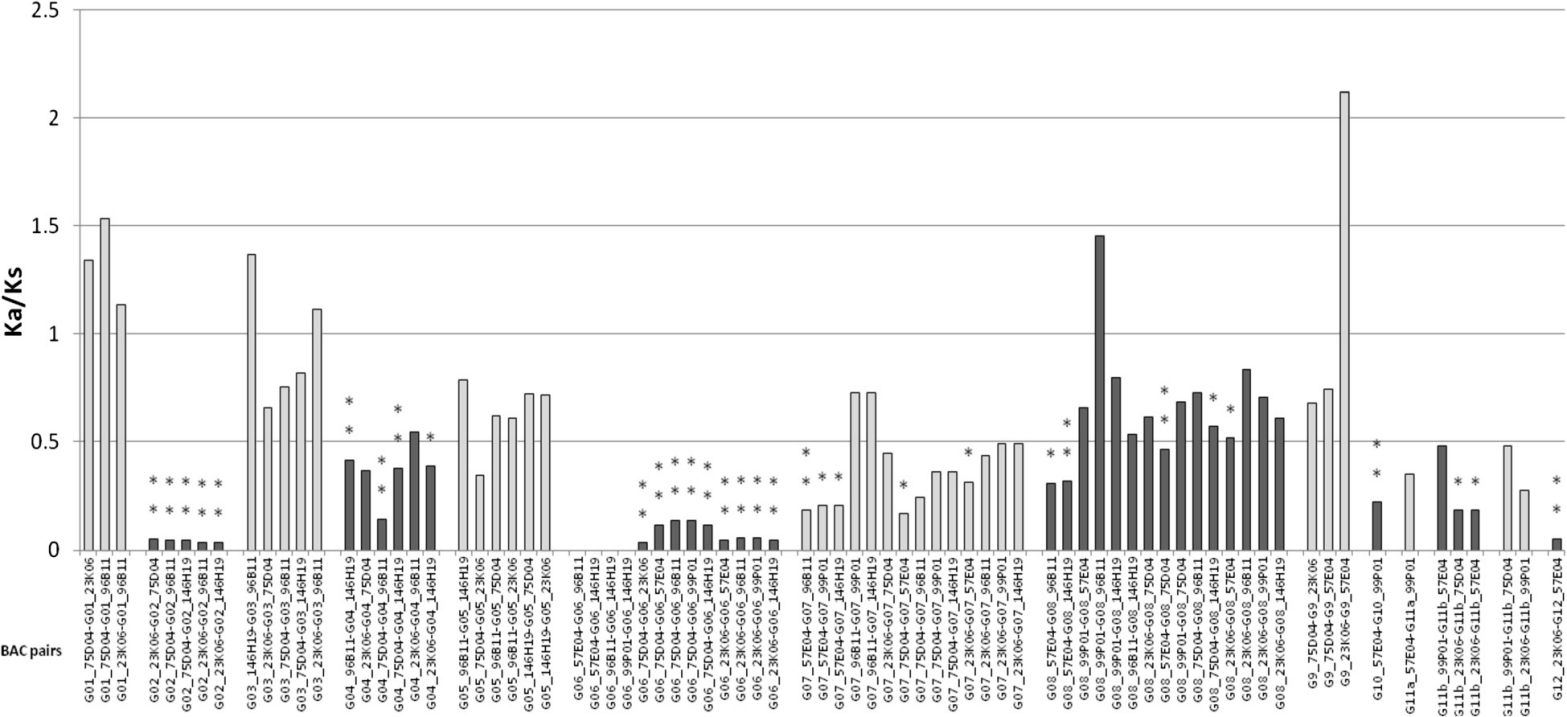
Ka/Ks for each gene from homolougs haplotype from *S.officinarum* (LA Purple) and *S.spotaneum*(AP85-441). Pairwise comparisons with Duncan’s test gave P values of *, P ≤ 0.05; **,P ≤ 0.01; ***, P ≤ 0.001

The Ka/Ks ratio of 12 pairs of genes was compared between the haplotypes of the two *Saccharum* species and *Saccharum* hybrids to estimate the selective constraints for the homologs (Additional file 7). In comparison, no significant difference of Ka/Ks ratios were observed in the homologous genes among *Saccharum* species (Additional file 8). Among the 13 unique genes, 451 gene pairs from *Saccharum* species, *Saccharum* hybrids and *Sorghum* were used for Ka/Ks analysis. The Ka/Ks ratios of 428 gene pairs (94.5 %) were less than 1 while the Ka/Ks ratio of the rest 23 gene pairs was above 1. These results suggested that the majority of homologous alleles were under purifying selection. Gene pairs of 11b in *S. spontaneum* haplotypes were found to under stronger purifying selection than in all the gene pairs in both *S. officinarum* haplotypes in LA Purple and *S. officinarum-*origin haplotypes in the hybrid, which might reflect the reduced functional selective constraint in *S. officinarum* caused by the duplication of the gene 11a (Fig. 3 and Additional file 7). However, the Ka/Ks ratios of gene 11a’s pairs were very low in *S. officinarum* haplotypes, indicating that the gene 11a contributes more critical function to *S. officinarum* than gene 11b. The gene pairs of gene 2, 6 and 12 in haplotypes of *S. officinarum* and *S. officinarum-*origin in hybrid had a Ka/Ks less than 0.5, indicating strong selective constraint and their critical function for *S. officinarum*. (Fig. 3 and Additional file 7).

### Neutrality test

To investigate whether the homologous haplotypes fit the neutral equilibrium model, we performed Tajima’s D test for 4 *S. spontaenum* (origin) haplotype sequences and 8 *S. officinarum* (origin) haplotype sequences, respectively. A significant negative Tajima’s D-test statistic indicates an excess of the low frequency of polymorphism, which is consistent with directional selection or population expansion [56]. We observed significant negative Tajima’s D value (−1.00205; *P* < 0.001) in the homologous regions from *S. spontaenum*, suggesting these regions were under directional selection. While, no significant negative Tajima’s D value (average = −0.67355, P > 0.1) was observed in the haplotypes from *S. officinarum* (Fig. 5). In addition to Tajima’s D, nucleotide diversity (Pi value) was estimated using DnaSP 5.0. We observed significant nucleotide diversity between *S. spontaneum* and *S. officinarum* (mean = 0.38 versus mean = 0.54, Mann–Whitney-Wilcoxon test, *P* value < 2.2 x 10–16). More than 60 % of nucleotide diversity for *Bru1* lost in the genomic regions of *S. spontaneum*, whereas *Bru1* in *S. officinarum* kept relatively higher DNA diversity. Fu and Li’s D test analysis showed similar results. A negative D value (−0.54) was observed in *S. spontaneum*. Meanwhile, sliding window (window size 1 kbp and step length 100 bp) showed that D values of a number of windows ranging from 11800 to 20300 were significant in S. spontaneum (P value < 0.01). However, a positive D value (0.12269) of F and Li was found at *Bru1* region in S. officinarum. These results were consistent with directional selection of *Bru1* in S. spontaneum.

### Comparison of large TEs between the homologous regions of hybrid R570 and its progenitor genome

The sugarcane hybrid R570 BAC sequences have approximately 10–15 % higher interspersed repeat content (70.0 %) than in the *S. officinarum* (59.0 %) and *S. spontaneum* (55.7 %) BAC sequences (Table 2), which is mainly due to higher Type I transposable element content in R570 than that in *S. officinarum* and AP85-441 BAC sequences. The AP85-441 BAC sequences have an exceptionally low amount of gypsy type LTR retrotransposons (4.4 %) (Table 2)*.* The *Saccharum* hybrid R570 BAC sequences have higher content of gypsy type elements (21.12 %) than copia (11.61 %), unlike *S. spontaneum* and *S. officinarum* BAC sequences that have higher content of copia-type elements (16.24 % and 18.92 % respectively) than gypsy (4.37 % and 12.06 %) (Table 2). Additionally, hybrid R570 BAC sequences have more LINE elements (7.32 %) than *S. spontaneum* (4.78 %) and *S. officinarum* BAC sequences (1.79 %) (Table 2).

The type II TE content in the *Bru1* genomic region of the sugarcane hybrid R570 is comparable to its progenitor genomes, though the content of individual families differs. For example, the hybrid R570 BAC sequences have 2.4–2.5 fold lower PIF-Harbinger transposons and 3 to 4 fold higher CACTA/CMC-EnSpm transposons than the sequences of two progenitors. The AP85-441 BAC sequences have about 2.2–2.6 fold higher Tc1-Mariner transposons than in *S. officinarum* and hybrid R570 BAC sequences, reflecting the differential accumulation of particular TE subfamilies in *S. officinarum*, *S. spontaneum*, and the hybrid.

**Fig. 4:**
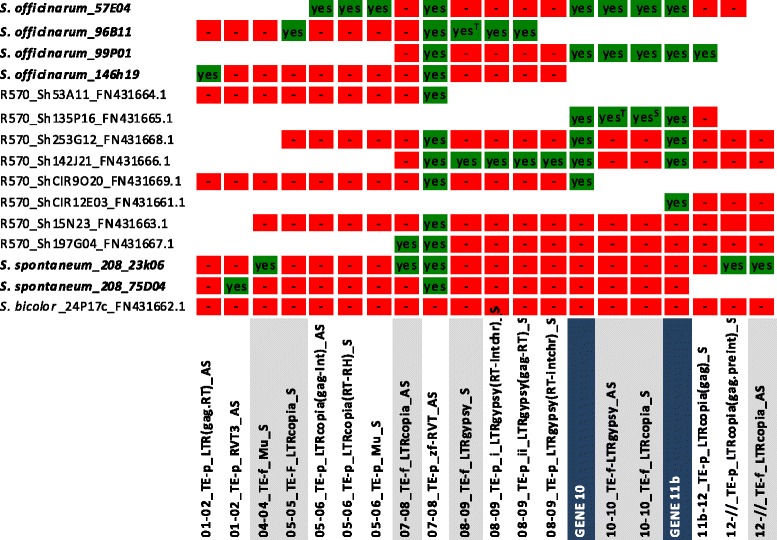
Distribution of transposable elements (TE) in the *Bru*1 surrounding regions of LA Purple (*S.officinarum*), AP85-441 (*S. spontaneum*), and the hybrid cultivar, R570. Notes: The TE names listed in the bottom row consist of four parts separated by an underscore” –“. The first part indicates the location, e.g., 01–02 indicates between gene 1 and gene 2 and 05–05 indicates within gene 5. The second part indicates full length (TE-f) or partial (TE-p). The third part indicates orientation (AS = Antisense, S = Sense). Colum 1 contains the clone identifiers: the species name, BAC name, and accession (if any) separated by underscore and the remaining columns score the presence (green) or absence (red) of each TEs listed at the bottom. A superscript “T” indicates a likely full length TE that was truncated due to its presence at the end of BAC and a superscript “S” indicates a solo LTR. The presence and absence of gene 10 (dark blue highlight in bottom row) and gene 11b (dark blue highlight) is also given for reference because these two genes are present in S*. officinarum* BAC sequences but absent in S. s*pontaneum* BAC sequences. The blank cells in white indicate no data available

To study the evolution of the *Bru1* locus, the large TEs in the *S. officinarum* and *S. spontaneum* BAC sequences at the *Bru1* genomic region were assessed in comparison with those in the corresponding hybrid R570 BAC sequences (Additional file 6). Four full-length Ty1/copia elements, 2 full-length Ty3/gypsy elements, one full-length Mu-like element, and 10 partial elements (9 retrotransposons and 1 transposon) in the *S. officinarum* and *S. spontaneum* BAC sequences were identified (Fig. 4)

The most conserved TE in the *Bru1* region is a partial Zn-finger domain (~38 aa homology to pfam13966: zf-RVT) located approximately 102 nucleotides downstream of the coding region of gene 8 (a conserved hypothetical protein). The universal presence of this domain is not only reminiscent of the shared ancestry of this region but is also suggestive of co-option of this derived segment in gene 8. This is supported by the fact that gene 8 mRNA from sorghum (GenBank accession number XM_002453182.1) includes part of this Zn-finger domain.

*S. spontaneum* clone Ss-23 k06 and hybrid R570 clone Sh197G04 share a retroelement (full-length in both BACs) located between genes 7 and 8. *S. officinarum* clone So-96B11 and R570 hybrid clone Sh142J21 share 3 retroelements (2 partial TEs and one TE full-length in *S. officinarum* but truncated in hybrid R570 due to its location at the end of BAC) located between genes 8 and gene 9. *S. officinarum* clones So-57E04 and So-99P01 and the R570 hybrid clone Sh135P16 share two nested TEs (full-length in both of the *S. officinarum* clones but truncated in the clone from hybrid R570 due to its location at the end of BAC) within the gene 10. R570 clone from *Bru1* genomic region was classified into 7 haplotypes [18]. Our results suggest that R570 BACs, Sh142J21 (haplotype V), Sh135P16 (haplotype VI), Sh197G04 clone (haplotype III) were evolved from *S. officinarum* haplotype, So-96B11, *S. officinarum* haplotype So-57E04 (and/or possibly So-99P01), and *S. spontaneum* haplotype Ss-23 K06, respectively. In addition to the shared TEs, we identified three full-length TEs and seven partial TEs in *S. officinarum* or *S. spontaneum* that were not detected or lost at the corresponding location in the hybrid R570 BACs.

Of the nine full-length retrotransposon insertions in the *Bru1* locus, six are estimated to be inserted at approximately 1 MYA (ranging from 0.88 to 1.28 MYA), and two were relatively young (inserted 0.00 Ma and 0.02 Ma) (Additional file 9). Surprisingly, the insertion time of Ty3/gypsy element (0.44 Ma) within gene 10 of *S. officinarum* So-99P01 sequence is much shorter than the insertion time of a Ty1/copia (0.99 MYA) nested within this one and also much lower than its counterpart (1.00 MYA) in the other *S. officinarum* BAC, So-57E04 (*Fig.*4). Both TEs in gene 10 of So-99P01 are flanked by intact target site duplications (TSD) and have dispersed mismatches in their respective pairs of LTRs, precluding mis-assembly or localized sequencing errors. Thus, either chance or other factors such as gene conversion may have played a role in the sequence preservation of LTR of this Ty3/gypsy element.

## Discussions

Modern sugarcane cultivars are developed from hybridization between *S. officinarum* with high sugar content and *S. spontaneum* with stress tolerance. Limited genetic diversity of parental clones became the bottleneck for modern sugarcane breeding. Identification of haplotypes of the main *Saccharum* species and tracing their evolutionary history after hybridization will provide essential information for sugarcane improvement. The isolation and sequencing of BACs in the genomic regions of the rust resistance gene in *S. officinarum* and *S. spotaneum* offered an opportunity to study the genomic features of the progenitor species in these fast-evolving and agronomically important sequences, and to validate the prediction of haplotype origins in hybrid R570.

Although haplotypes were highly conserved within and between *Saccharum* species, our study identified species-specific insertions and deletions, which likely occurred after the speciation event, and can be used to identify origins of haplotypes in modern sugarcane hybrids. Interestingly, genes 10 and 11a were completely missing in *S. spontaneum*, which might attribute to the consequence of the speciation event. Multiple alleles in autopolyploids reduced selective constraint for those genes with no advantage in higher dose, and some alleles could have undergone pseudogenization. In the genomic region of rust resistant genes, 6 out of 52 gene alleles became pseudogenes. Out of the 15 paired alignments, InDels broke down alignments in 32.18 % sequences, which caused a frame shift and introduced premature stop codons in some alleles and made them pseudogenes.

Comparison of haplotype sequences within species showed that *S. spontaneum* had larger haplotype variations than that of *S. officinarum* (Additional file 10), suggesting earlier polyploidization in *S. spontaneum* than in *S. officinarum*, which could have contributed to or even caused the speciation event leading to the divergence of *S. spontaneum* from the rest of *Saccharum* species. In general, the sequence divergence is the highest between *S. spontaneum* and *S. officinarnum*, medium among *S. spontaneum* haplotypes, and the lowest among the *S. officinarnum* haplotypes.

*Saccharum* species had undergone extensive genome rearrangements following polyploidization in the *Bru1* region, which is similar to the instability of maize genome after polyploidization [57]. Gene 11a and gene 11b are duplicated genes in *S. officinarum*. Gene11b existed in all of the *S. officinarum* haplotypes but was missing in *S. spontaneum* and sorghum (Fig. 1 and Table 5). Phylogenetic analyses showed that gene 11b was closer to its homologs in sorghum and rice than the 11a observed in *S.officinarum* (Additional file 11), which indicated a duplication event of gene 11 occurred after the speciation event separating *Saccharum* and *Sorghum*. Due to the absence of gene 11b in *S. spontaneum* genome, the duplication event of gene 11 likely occurred after the speciation event of *S. officinarum* and *S. spontaneum* and could be lineage specific in *S. officinarum*.

Comparing to *Bru1* region in *Saccharum* species, no large TEs were observed between the genes of the corresponding region in sorghum (Fig. 4). The large TEs between genes in *Adh1* region of *Saccharum* hybrids were also absent in the corresponding region of sorghum genome [58]. A TE zf-RVT in the *Bru1* regions of *Saccharum* was speculated to derived from gene 8 (Fig. 4), indicating small fragment duplication occurred before the polyploidization of *Saccharum* and after the separation between *Saccharum* and *Sorghum*. Moreover, TEs are more abundant in *S. officinarum* (59.04 %) than in *S. spontaneum* (55.68 %) (Table 2). These results suggested that genome expansion in this region of *S. officinarum*, compared to S. *spontaneum*, was caused by TE accumulation.

Modern sugarcane hybrids contain estimated 8–14 copies of homologous chromosomes, and can have up to 14 different alleles [59]. Although multiple alleles are considered to be functionally redundant at the time of origin, they provide raw materials for the evolution of novelty by relaxing purifying selection on the duplicated genes [60–63]. Six out of 51 allelic genes became pseudogenes in *Saccharum*, likely due to functional redundancy. *S. spontaneum* genome had undergone more dynamic genome rearrangement than *S. officinarum* genome. In paleopolyploids [64–67], and recent allopolyploid species, such as wheat [68, 69] and Tragopogon [70, 71], eliminations and pseudogenizations of essential functional genes have been well documented.

Among the 13 unique genes, 451 allele pairs from *Saccharum* species, *Saccharum* hybrids and sorghum were used for Ka/Ks analysis, excluding genes that were missing in the *S. spontaneum* haplotypes. The Ka/Ks ratio of 94.5 % allele pairs (428 pairs) was less than 1, suggesting that the majority of homologous alleles were under purifying selection, which is consistent with the Ka/Ks ratios of genes in haplotypes of hybrid R570 [18]. Selection resulted in nonrandom radical amino acid substitutions for many genes [72]. Our results verified that gene 11a was a *S. officinarum* specific gene and likely a recent duplication from gene 11b, resulting a lower Ka/Ks ratio.

Gene structures are highly conserved in *Bru1* and *Adh1* regions among haplotypes within the R570 genome [18, 58]. InDels were found in introns of genes when compared to sorghum and introns exhibited more variations than exons as expected [58]. In our study, except for genes 1, 2, and 9, the other 10 unique genes exhibited variable sizes of introns among the homologous alleles (Additional file 10), which are resources for developing intron length polymorphism markers in sugarcane. Variations in introns have been associated with biological function both in animal and plant [73, 74]. Insertions of LTRs were found in introns of genes 4, 10,11a and 11b, some of which could have evolved new functions via neofunctionalization, or partition their ancestral roles via subfunctionalization. Intron gain/loss events were found in hybrid R570, such as gene 1 in ShIV (Sh53A11), gene 8 in ShV (Sh15N23) (Fig. 4). Intron gain/loss is not a commonly ongoing process, but rather triggered by certain dramatic evolutionary events that lead to long-term bottlenecks [75]. Since intron gain/loss events were only observed in the hybrid genome, they might have been triggered by hybridization event.

To discriminate the origin of the genome in the hybrids, we performed three comparative analyses: sequence similarity, species-specific InDels, and gene phylogenetic combined with haplotype networks. Sequence comparison among the homologous haplotype could be used to identify the origin of most regions in *Saccharum* hybrids genome, but may not be sufficient to discriminate the recombinant haplotype due to the little divergence between *S. officinarum* and *S. spontaneum.* Species-specific InDels could be reliable markers for identifying the origin of *Saccharum* hybrid genome. Further comparison of genome between *S. officinarum* and *S. spontaneum* may identify the InDels for discriminating the recombinant genome in *Saccharum* hybrids. Gene phylogenetic and haplotype network analysis could be used for identifying recombinant genome in *Saccharum* hybrids(Fig. 2).

One of the major challenges for sequencing sugarcane hybrid cultivar genomes is to distinguish the fractions from *S. spontaneum*, *S. officinarum* and the recombinant genome. We performed the sequence analysis of homologous haplotypes from the progenitor species of *Saccharum* hybrids surrounding the *Bru1* region. Retrotransposon insertions and sequences variations among the homologous haplotypes sequence divergence ranged from 18.2 % to 60.5 % with an average of 33.7 %, comparable to the 12.8-23.3 % InDels divergence among homologous chromosomes in hexaploid wheat, which allows sequencing and assembling the autopolyploid *Saccharum* genomes and the auto-allopolyploid hybrid genomes using whole genome shotgun sequencing approach as demonstrated in wheat [76, 77]. However, long read sequencing would be necessary for discriminate the homologous haplotypes in the progenitor species because identical fragments of short reads exist in *Saccharum* genomes.

**Fig. 5:**
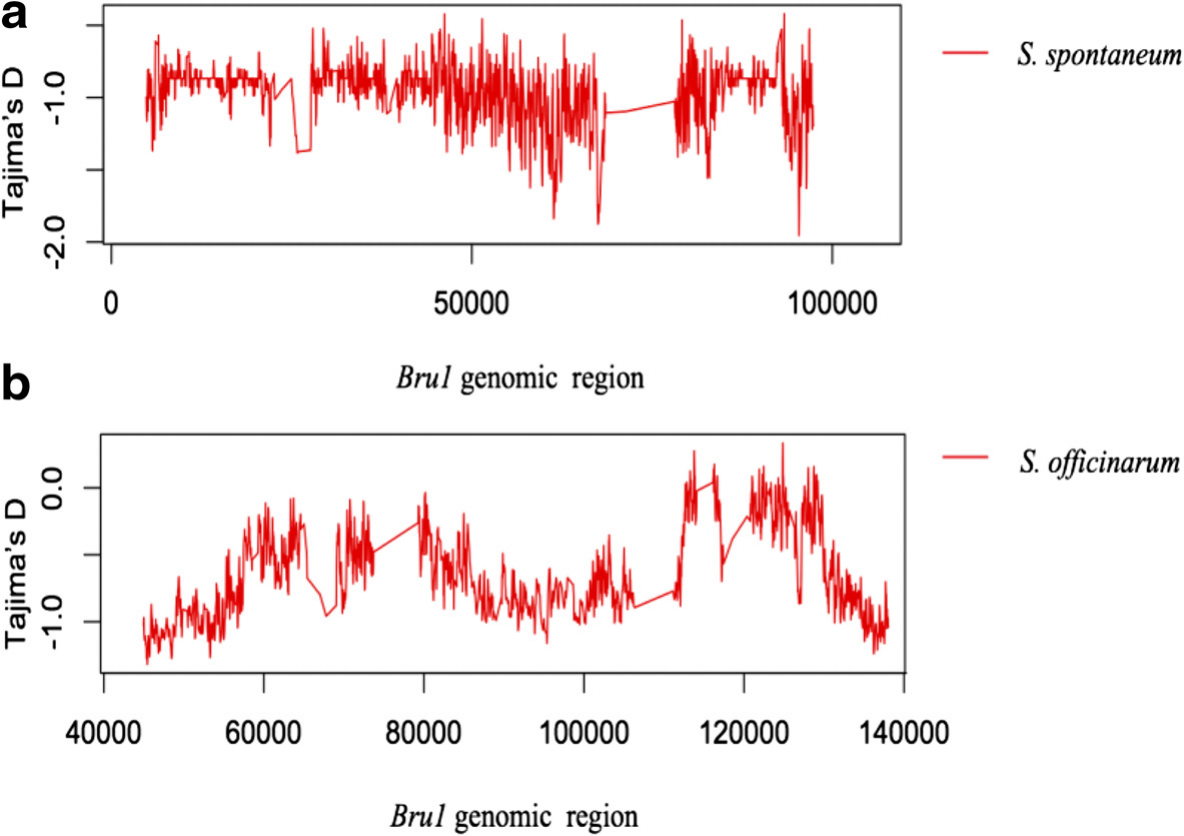
Tajima’D test for *bru1* genomic regions from *S. spontaneum* (**a**) and *S. officinarum* (**b**). Notes: Homologous haplotype sequences in *S. spontaneum* include Sh15N23, Sh197G04, Ss-75O04 and Ss-23 K06. Homologous haplotype sequences in *S. officinarum* contain So-96B11, So-146H19, So-57E04, So-99P01, Sh53A11, Sh135P16, Sh253G12 and Sh142J21

The genome sequence diversity in wild species *S. spontaneum* have been demonstrated to be greater than that of the domesticated species *S. officinarum* [78–81]. However, the two *S. spontaneum* homologous sequences (Ss-75D04 and Ss-23 K06) shared higher similarity(98 %) than the sequence similarities among the homologous haplotype sequences from *S. officinarum*.Moreover, *S. spontaneum-*originated hapotypes were under directional selection with an average windowed Tajima’s D value of −1.00205 (*p* < 0.001), while, the *S. officinarum* haplotypes showed no significant negative Tajima’s D value. *S. spontaneum* contributed stress tolerance for *Saccharum* cultivar hybrid (Fig. 5). These results suggested that the *Bru1* genomic region in *Saccharum* hybrid originated from *S. spontaneum* and is under strong directional selection. The candidate *bru1* gene is supposed to be under strong functional constraint and has a more substantial selection *in S. spontaneum than that in S. officinarum.* Of 10 homologus genes in the *Bru1* genomic haplotype regions*,* 5 (gene 2, 4, 6, 7 and 8) were revealed to under strong functional constraint based on the ka/ks analysis, but none of them were found to have a higher selection in *S. spontaneum* than in *S. officinarum*. The *bru1* gene in R570 was revealed to be single-dose [13], it is challenge to predicted dosage of *bru1* gene in S.spontaneum, but we can conclude that *bru1* is not octopi-dosage as the *bru1* in R570 was single-dose. Therefore, the *bru1* gene may not existed in the two haplotype sequences from *S. spontaneum* as only quarter of alleles were sequenced, but it could be presented in the *S.spontaneum* originated haplotype within R570 because 2 alleles from *S.spontaneum* may cover all the *S. spontaneum* originated alleles in the *Saccharum* hybrid. Map-based cloning is hard to refine the gene in such limited genomic region. To further identify the *bru1* gene, gene expression experiment based on RNA-seq could be used to test the expressional level of the functional constraint genes. Candidate gene transformation would be necessary to final confirm the *bru1* genes.

## Conclusions

The commercial sugarcane cultivars are complicated allo-autopolyploid and aneuploid, mostly derived from interspecific hybridization between *S. spontaneum* and *S.officinarum.* This study reports the first comparison among haplotypes of a modern sugarcane hybrid R570 and its progenitor species, and provides new insights into the genome evolution of modern sugarcane cultivars. With the homologous sequences from the two progenitor species as references, species-specific InDels were identified, which were used to validate the species origins of the 7 haplotypes from the hybrid genome of R570. Our results confirmed that 4 haplotypes of R570 were originated from *S. officinarum*, 2 from *S. spontaneum.* ShCIR9020-CIR12E03 was originated from recombination. Tajima’s D analysis showed that *S. spontaneum* haplotypes in *Bru1 genomic region* were under directional selection, contributing to brown rust resistance in *Saccharum* hybrid. The duplication event of gene 11 and InDels among the homologous haplotypes suggested that *Saccharum* species had undergone genome rearrangements after speciation. Gene content and gene structure were relatively well conserved among the homologous haplotypes. Exon splitting occurred in haplotypes of the hybrid genome but not in its progenitor genomes, signalling genome rearrangements after hybridization. Among all the homologous alleles, introns vary in size while the exons are conserved. Pseudogenes (alleles) caused by InDels were observed for all annotated genes except gene 10 in the two *Saccharum* species.

## Abbreviations

BAC, bacterial artificial chromosome; cM, centimorgan; TE, transposable element; SNP, single nucleotide polymorphism; InDels, insertion/deletion; MYA, million years ago; LTRs, long terminal repeat; RFLP, restricted fragment length polymorphism; MITE, miniature inverted transposable element; LINE, long interspersed element; CDD, conserved domain database; TSD, target site duplication

## Additional files

Additional file 1: Table S1. The sequences of primers used for probe preparation for the BAC library screening. (DOCX 15 kb) Additional file 2: Table S2. List of syntenic genes of the BAC clone sequences (DOCX 19 kb) Additional file 3: Figure S1. Pairwise comparision of BAC sequences from LA Purple (*S.officinarum*), AP85-441 (*S. spontaneum*), *Saccarhum* Hybrids(R570) (DOCX 497 kb) Additional file 4: Figure S2. Mauve visualization of local collinear blocks identified among 14 haplotypes (15BACs) from *Saccharum* species and sorghum. (DOCX 1909 kb) Additional file 5: Figure S3. Gene structure comparison of different haplotype sequences from LA Purple (*S. officinarum*), AP85-441 (*S. spontaneum*), and the hybrid cultivar, R570. (DOCX 700 kb) Additional file 6: Figure S4. The genome structure of haplotypes surrounding the *Bru1* locus from LA Purple (*S. officinarum*), AP85-441 (*S. spontaneum*), and the hybrid cultivar, R570. (DOCX 95 kb) Additional file 7: Table S3. Estimation of synonymous and non-synonymous nucleotide divergence among *S. officinarum* (LA Purple), *S. spontaneum*(AP85-441) and hybrid cultivar R570. (DOCX 24 kb) Additional file 8: Table S4. The average Ka/Ks ratio of the gene pairs within and between LA Purple (*S. officinarum*), AP85-441 (*S. spontaneum*), and the hybrid cultivar, R570. (DOCX 16 kb) Additional file 9: Table S5. Estimated insertion time of full length retrotransposons in *Bru1* locus of LA Purple (*S. officinarum*), AP85-441 (*S. spontaneum*), and the hybrid cultivar, R570. (DOCX 18 kb) Additional file 10: Table S6. Summary of the gapless comparisons of pairs of BAC clone sequences from LA Purple (*S. officinarum*), AP85-441 (*S. spontaneum*), and the hybrid cultivar, R570. (DOCX 39 kb) Additional file 11: Figure S5. Phylogenetic analysis of 14 haplotypes of genes 11 from *Saccharum* species, and its homologs from sorghum, *Zea mays,* and rice. The tree was constructed by the neighbor-joining method implemented in MEGA4 software. The robustness of the tree topology was assessed with 1000 bootstrap replicates. The coding sequences of S6PDH from *Zea mays* and *Malus domestica* were used to root the tree. (DOCX 108 kb)
