# Supplementary material for: Comparative structural analysis of Bru1 region homeologs in Saccharum spontaneum and S. officinarum
Source: BMC Genomics. 2016 Jun 10;17:446. doi: 10.1186/s12864-016-2817-9 (PMC4902974; doi:10.1186/s12864-016-2817-9)
Supplement: Additional file 1: Table S1. — The sequences of primers used for probe preparation for the BAC library screening. (DOCX 15 kb) [file 12864_2016_2817_MOESM1_ESM.docx]

Additional file 1:Table S.1. The primers of probes for BAC hybridization

| Probe name | Forward | Reverse | Produce Length(bp) | notes |
| --- | --- | --- | --- | --- |
| 8-CHP | CCAGCTCATCGTTTGCTACG | CTGCTGCTGAGACTAATCTTCCT | 549 | Gene 8 of FN431663 |
| 11b-NADP | CACTCGCAGAGGCGTTTCAGA | CCTTGCAGGCTTCAAGCACAT | 540 | Gene 11 of FN431663 |
