# Supplementary material for: Comparative structural analysis of Bru1 region homeologs in Saccharum spontaneum and S. officinarum
Source: BMC Genomics. 2016 Jun 10;17:446. doi: 10.1186/s12864-016-2817-9 (PMC4902974; doi:10.1186/s12864-016-2817-9)
Supplement: Additional file 2: Table S2. — List of syntenic genes of the BAC clone sequences (DOCX 19 kb) [file 12864_2016_2817_MOESM2_ESM.docx]

| Additional file 2:Table S.2 List of syntenic genes of the BAC clone sequences | | |  |  |
| --- | --- | --- | --- | --- |
| Gene NO. | Predicted gene | BLAST HIT | E-Value | orthologous sorghum locus |
| 1 | hypothetical protein (CHP) | hypothetical protein OsI_24617 [Oryza sativa Indica Group]EAZ02513.1 | 1.00E-31 | Sb04g001890 |
| 2 | histone lysine N-methyltransferase ATXR6 | Select seq ref\|XP_004952071.1\|  PREDICTED: histone-lysine N-methyltransferase ATXR6-like [Setaria italica] | 0 | Sb04g001900 |
| 3 | arginine ⁄ serine-rich splicing factor | arginine/serine-rich splicing factor RSP41 [Zea mays]( NP_001147698.1) | 6.00E-176 | Sb04g001910 |
| 4 | dimethyladenosine transferase | Select seq ref\|AFW66605.1\| dimethyladenosine transferase [Zea mays] | 0 | Sb04g001913 |
| 5 | Ulp1 protease family, C-terminal catalytic domain containing protein (Q2QME9) 1.E-142 | Select seq ref\|XP_006664726.1\|PREDICTED: probable ubiquitin-like-specific protease 2B-like [Oryza brachyantha] | 1.00E-95 | Sb04g001916 |
| 6 | putative cyclin-dependent kinase A family protein | Select seq gb\|AFW66610.1\|  putative cyclin-dependent kinase A family protein [Zea mays] | 0 | Sb04g001920 |
| 7 | shrunken seed protein (Q85851) 5.E-88 | Select seq ref\|XP_004952062.1\|PREDICTED: peroxisome biogenesis protein 16-like isoform X3 [Setaria italica] | 0.00E+00 | Sb04g001930 |
| 8 | conserved hypothetical protein (CHP) PO575F10.13 | Select seq ref \|AFW69903.1.\|hypothetical protein ZEAMMB73_535480 [Zea mays] | 0 | Sb04g001940 |
| 9 | tyrosine-specific protein phosphatase-like | Select seq gb\|EMT21109.1\|  Putative tyrosine-protein phosphatase [Aegilops tauschii] | 5.00E-11 | N/A |
| 10 | serine ⁄ threonine protein kinase pelle fragment | Select seq gb\|ABN45791.1\|  serine/threonine protein kinase [Hordeum vulgare subsp. vulgare] | 9.00E-40 | N/A |
| 11a | NADP-dependent D-sorbitol-6-phosphate dehydrogenase | Select seq gb\|BAD07953.1\| putative NADPH-dependent mannose 6-phosphate reductase [Oryza sativa Japonica Group] | 0 | Sb04g001950 |
| 11b | NADP-dependent D-sorbitol-6-phosphate dehydrogenase | Select seq gb \|NP_001149399.1\|PREDICTED: NADP-dependent D-sorbitol-6-phosphate dehydrogenase-like [Setaria italica] | 0 | Sb04g001950 |
| 12 | Endoglucanase 4 precursor | Select seq ref\|XP_003576683.1\|  PREDICTED: endoglucanase 4-like [Brachypodium distachyon] | 0 | Sb04g001960 |
