# Supplementary material for: Comparative structural analysis of Bru1 region homeologs in Saccharum spontaneum and S. officinarum
Source: BMC Genomics. 2016 Jun 10;17:446. doi: 10.1186/s12864-016-2817-9 (PMC4902974; doi:10.1186/s12864-016-2817-9)
Supplement: Additional file 3: Figure S1. — Pairwise comparision of BAC sequences from LA Purple (S.officinarum), AP85-441 (S. spontaneum), Saccarhum Hybrids(R570) (DOCX 497 kb) [file 12864_2016_2817_MOESM3_ESM.docx]

| 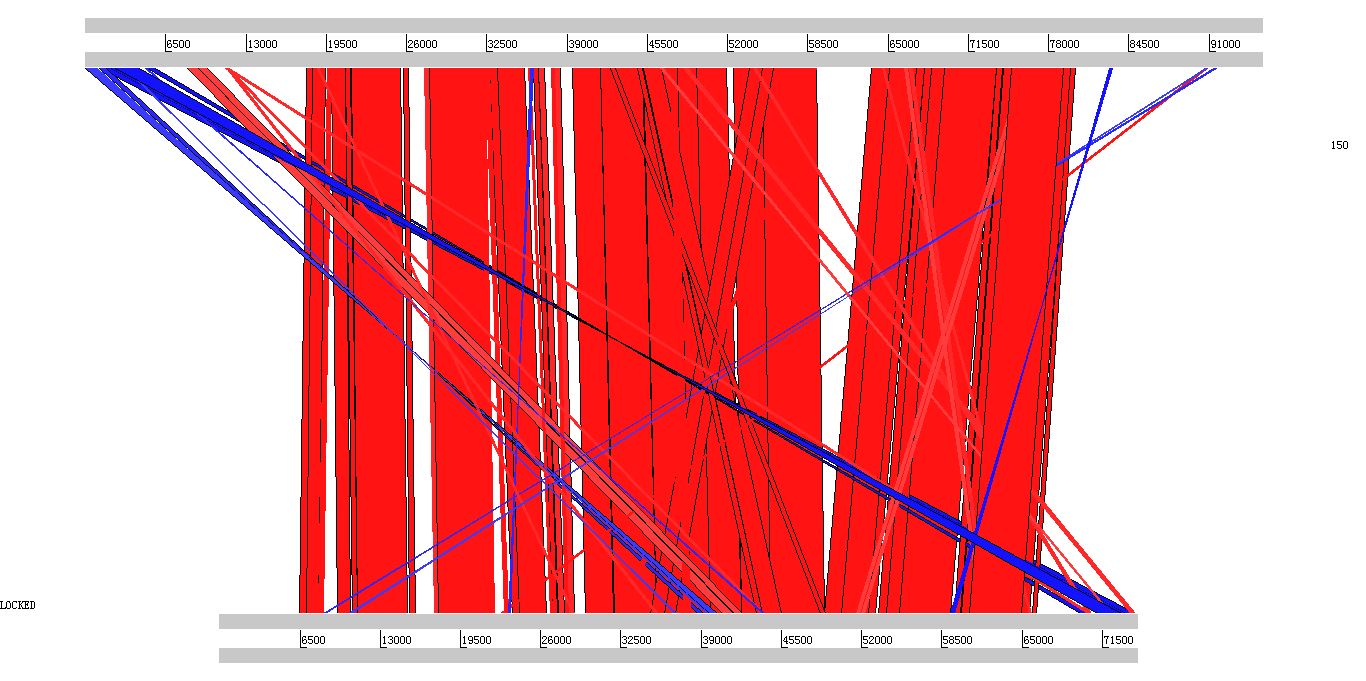  S1-1 | 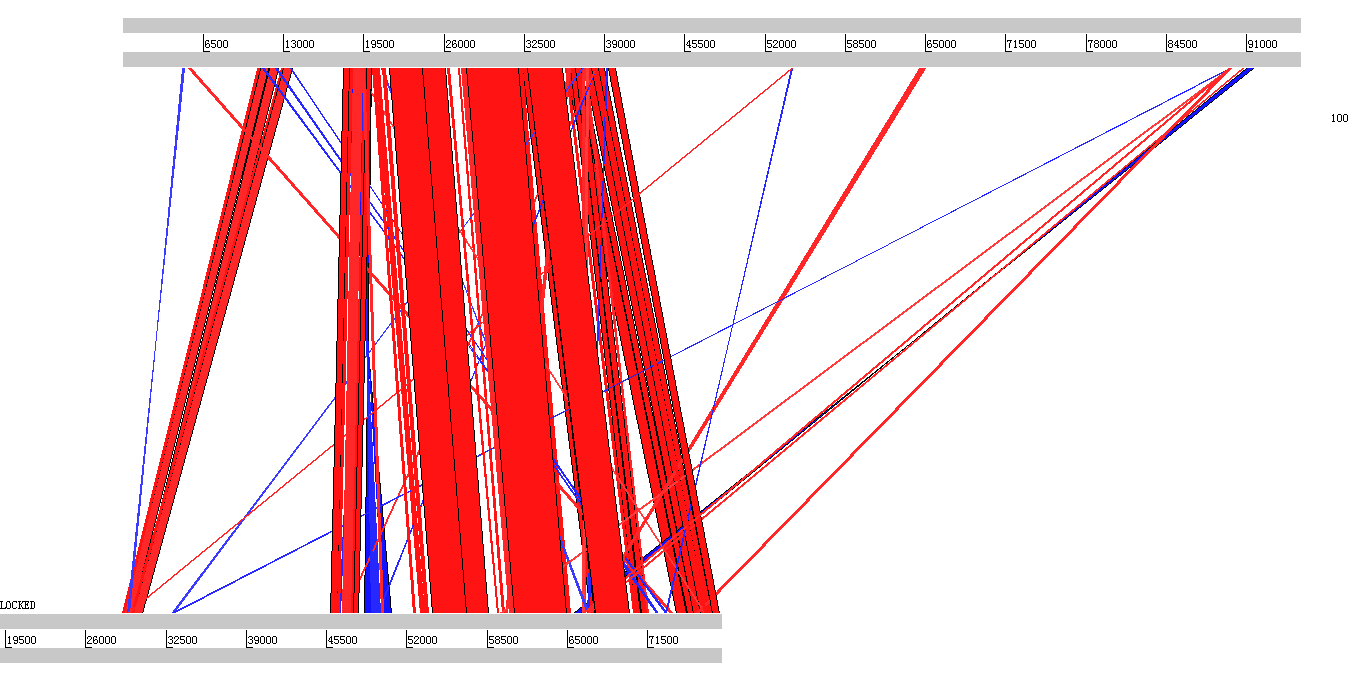  S1-2 | 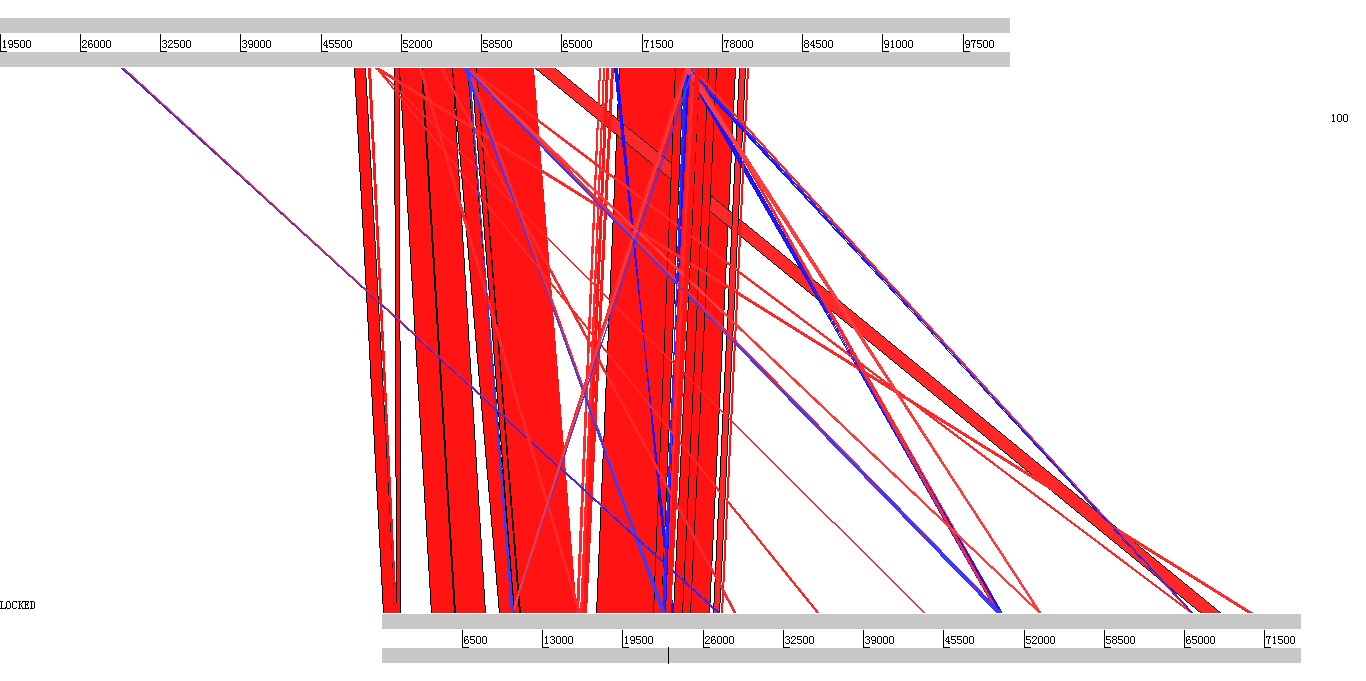  S1-3 |
| --- | --- | --- |
| 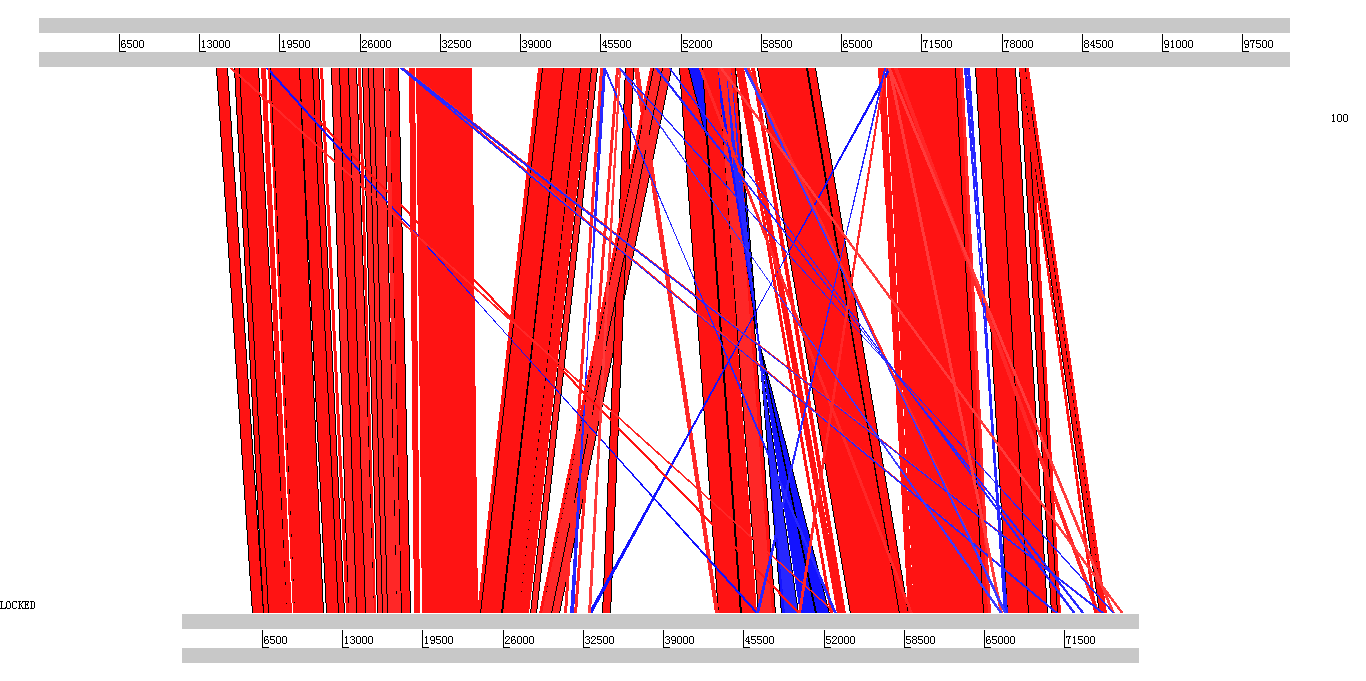  S1-4 | 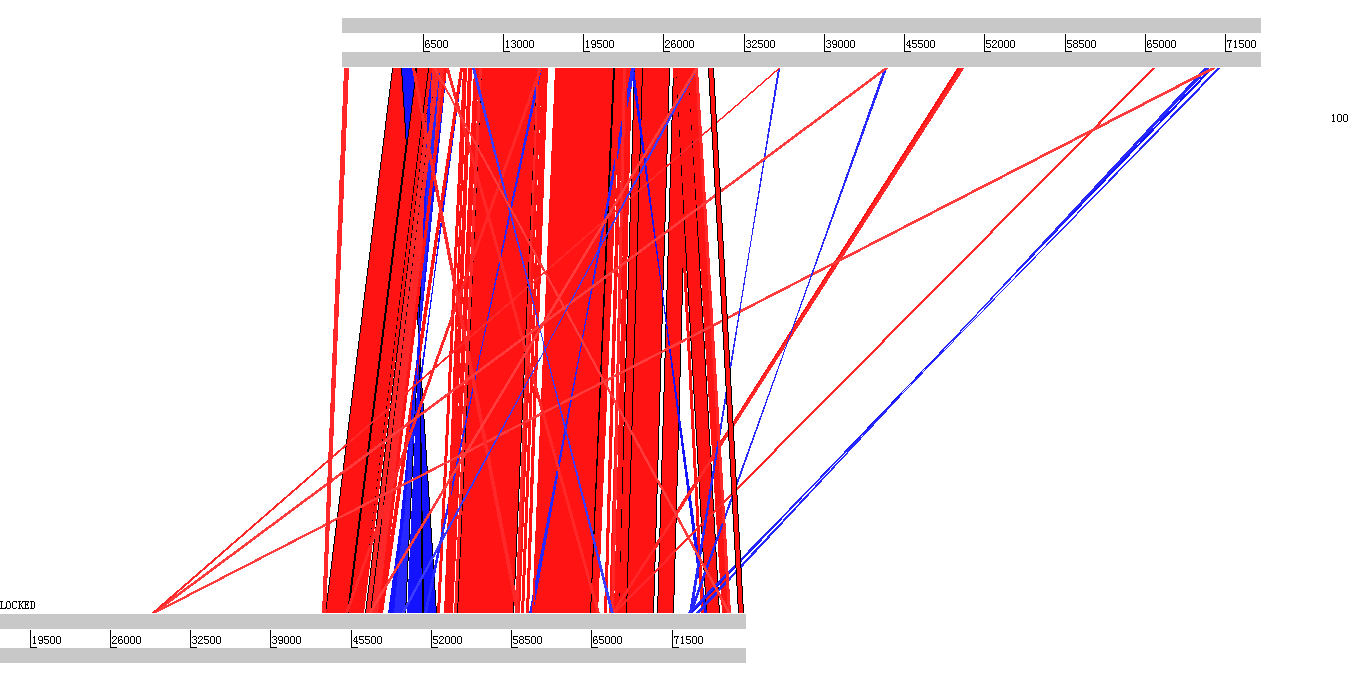  S1-5 | 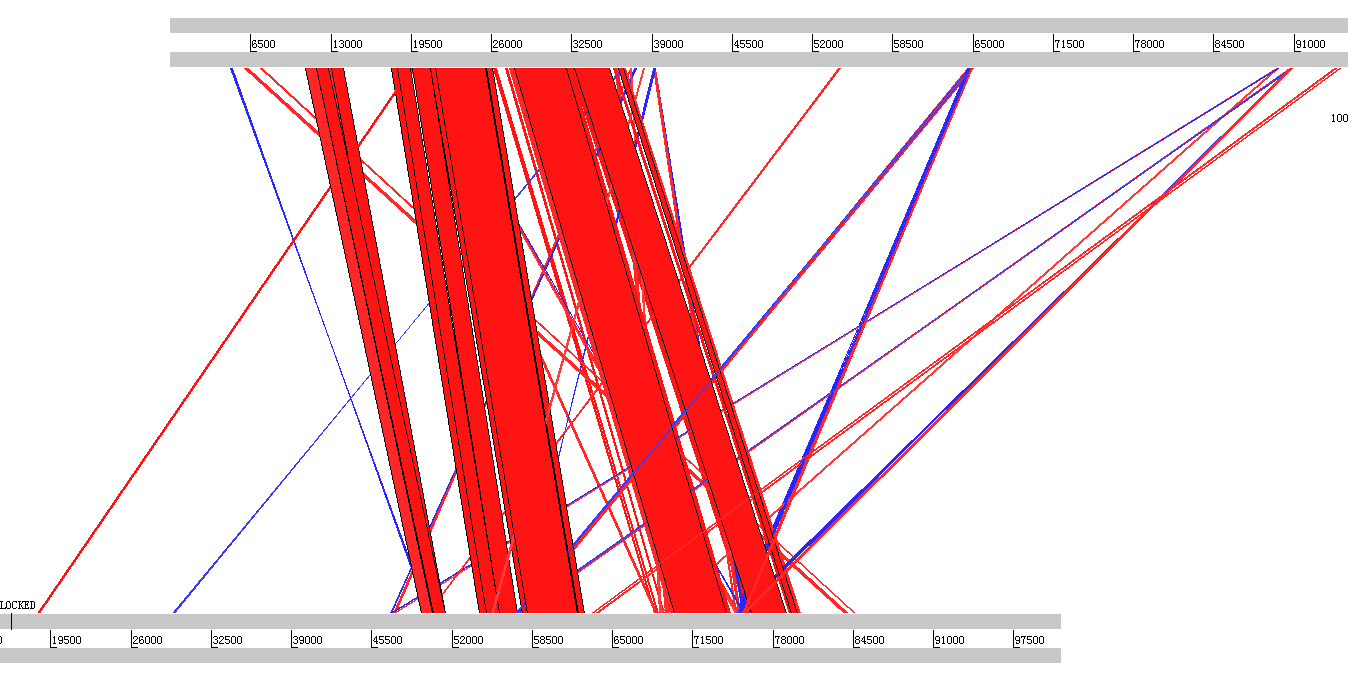  S1-6 |
| 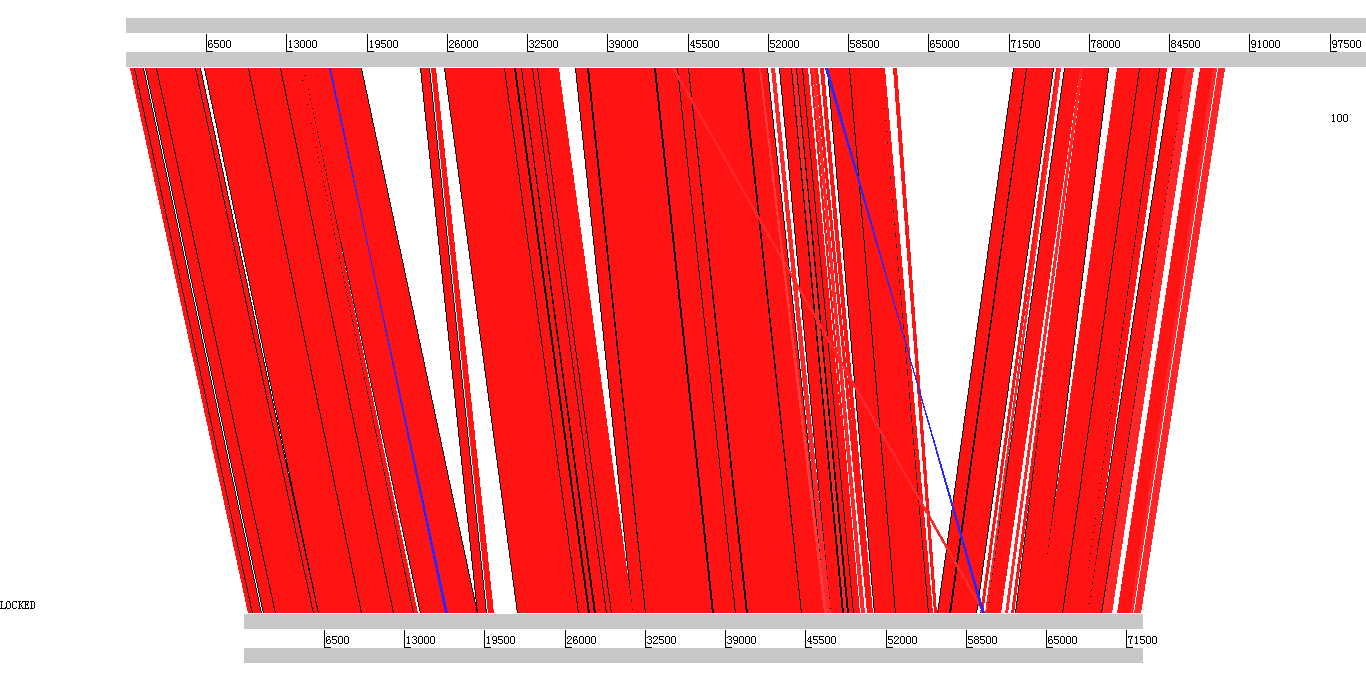  S1-7 | 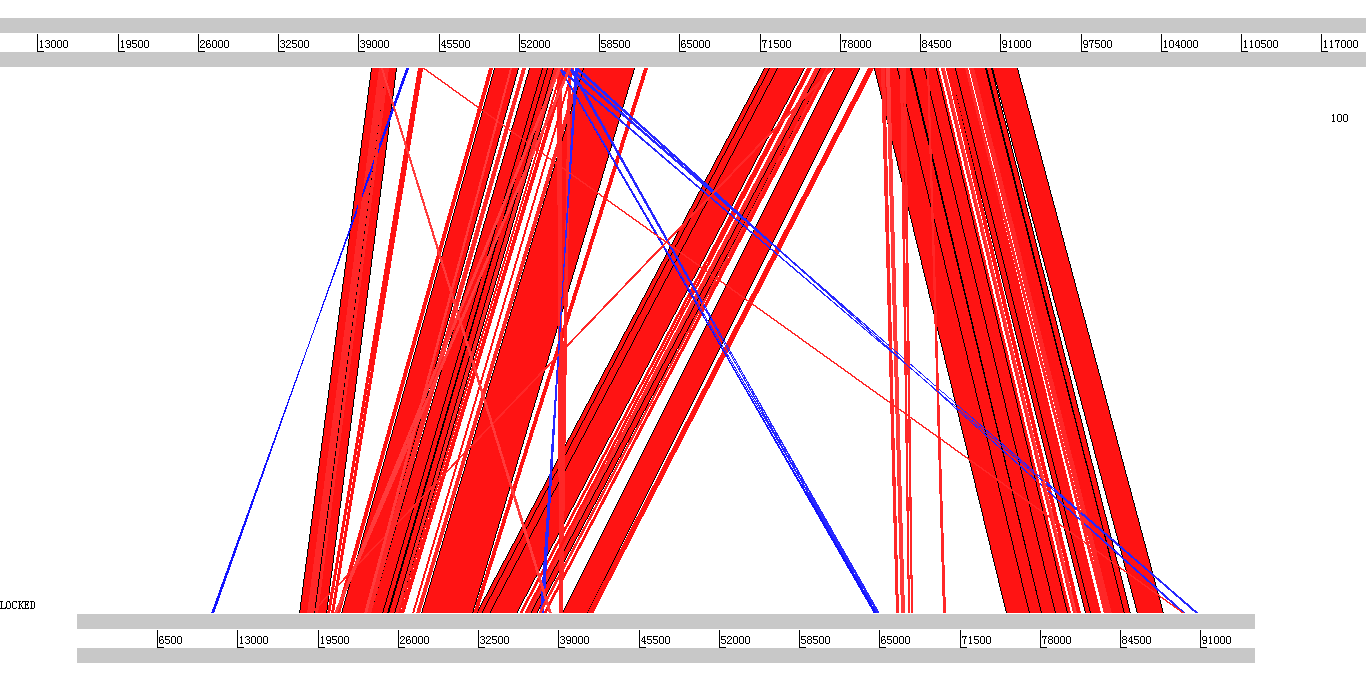  S1-8 | 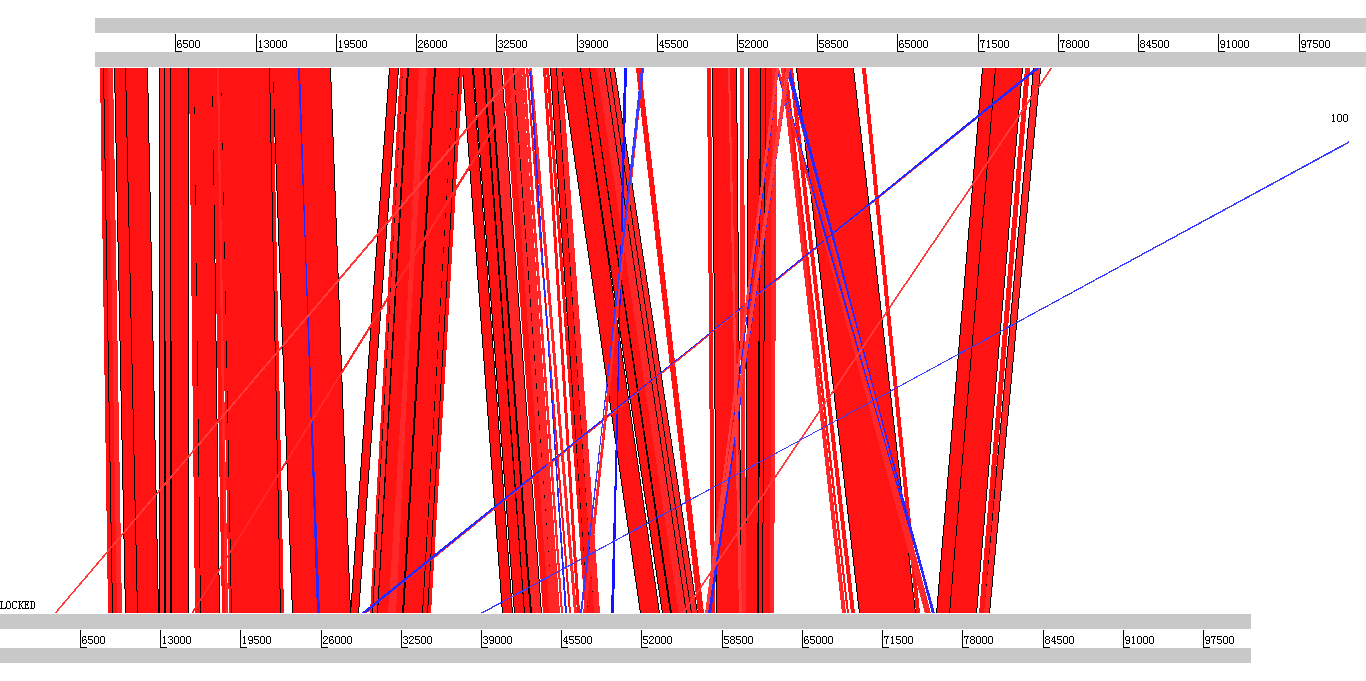  S1-9 |
| 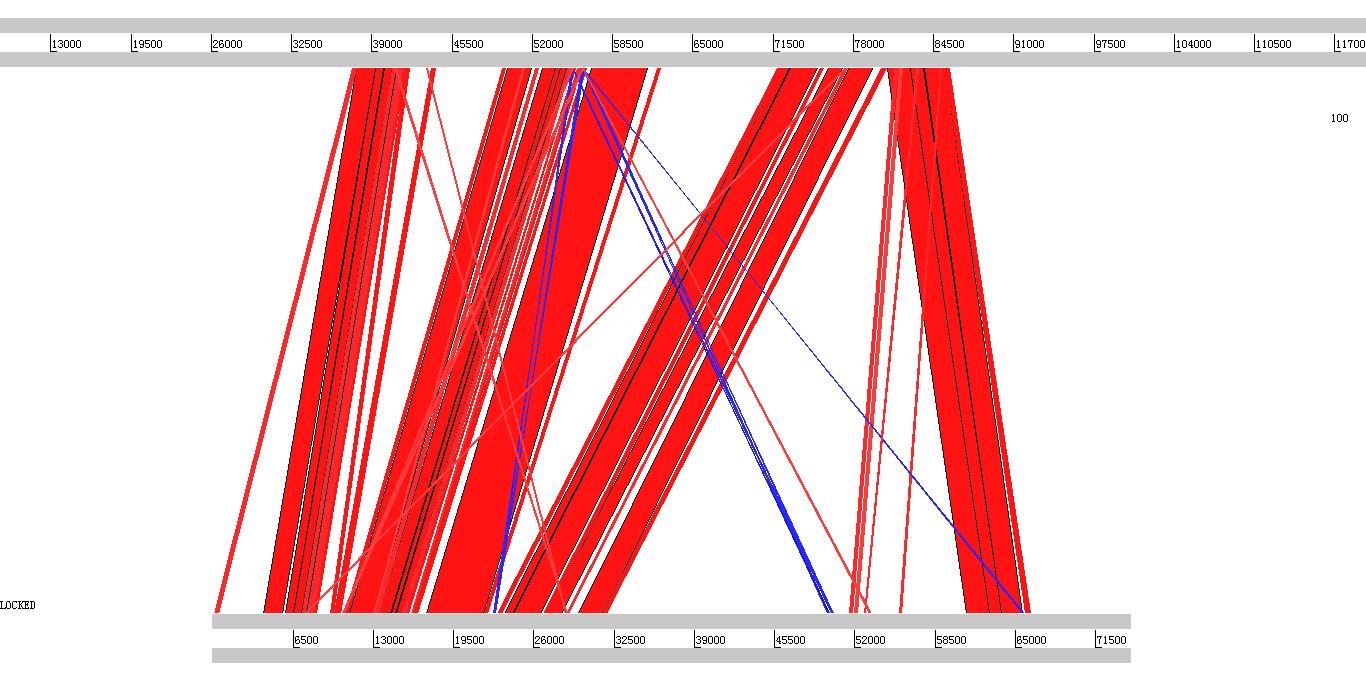  S1-10 | 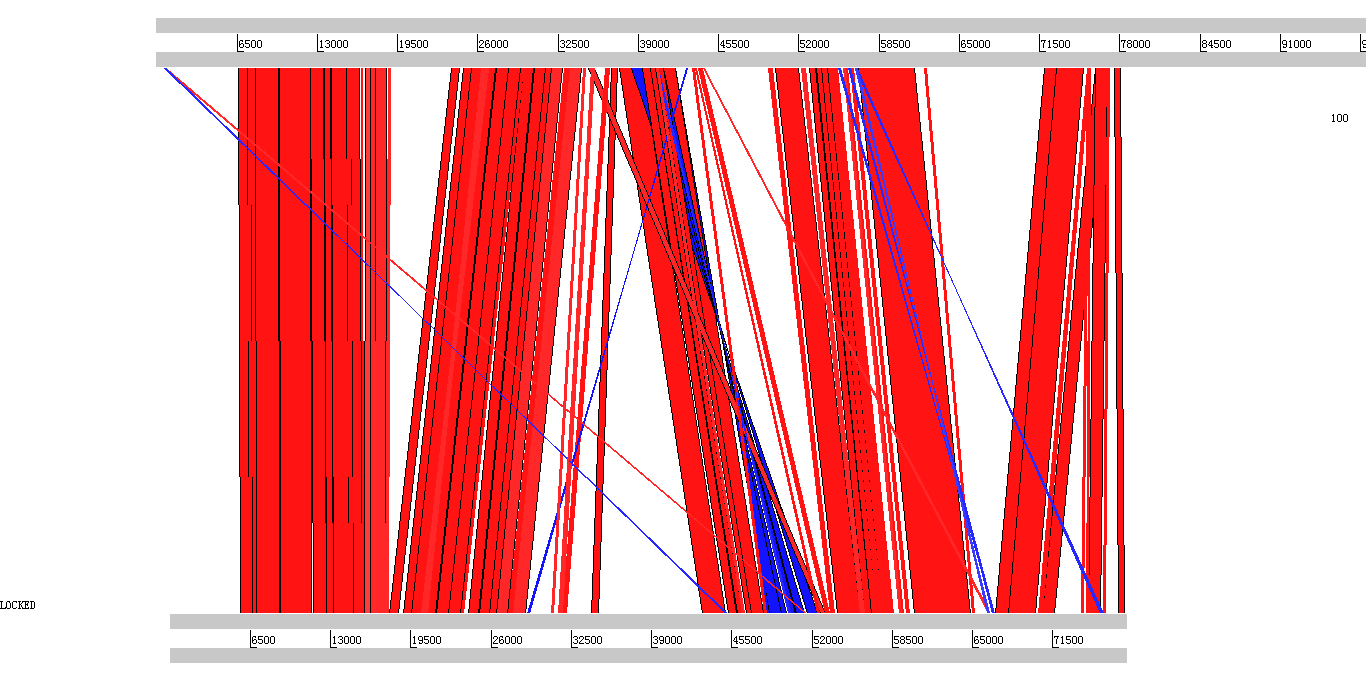  S1-11 | 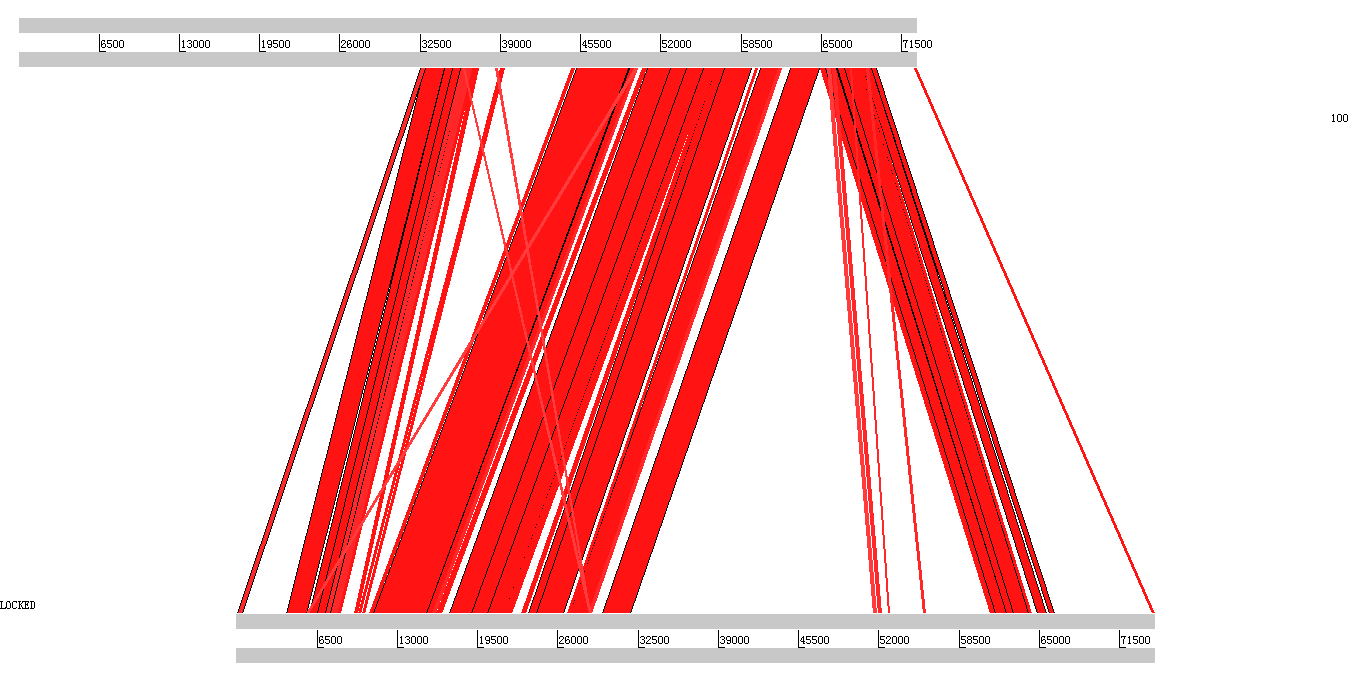  S1-12 |
| 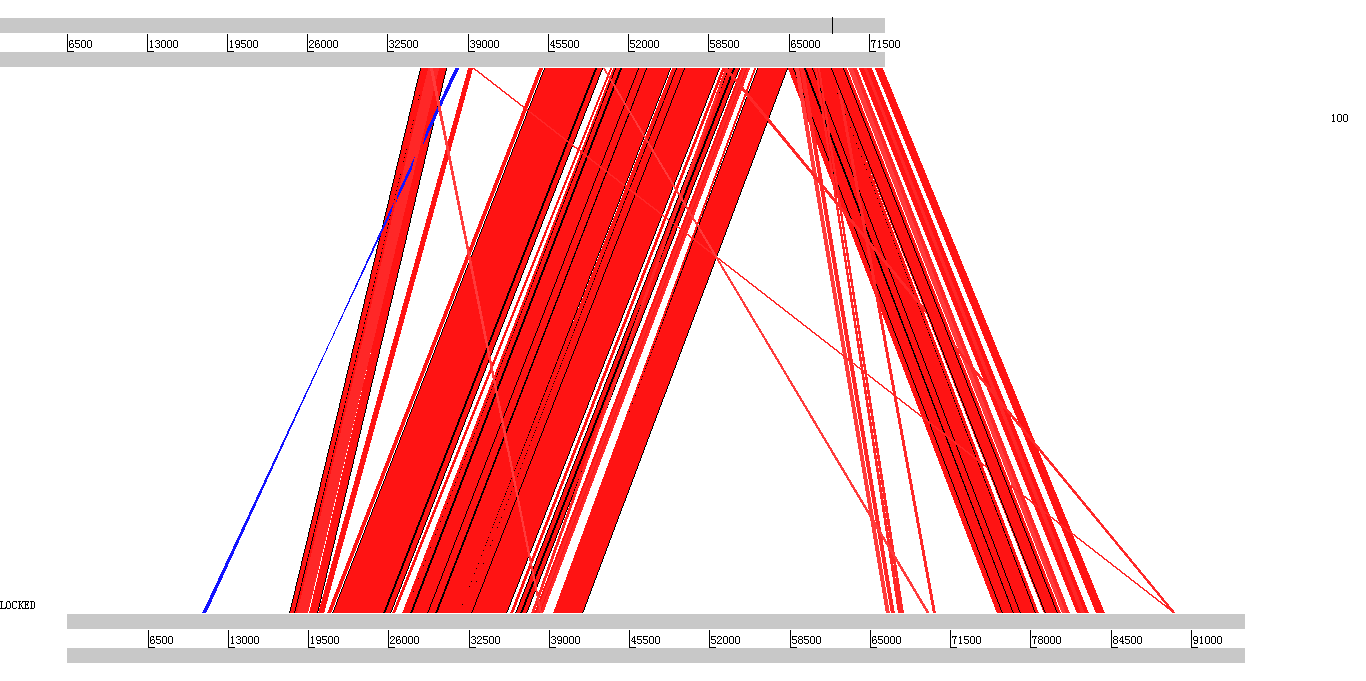  S1-13 | 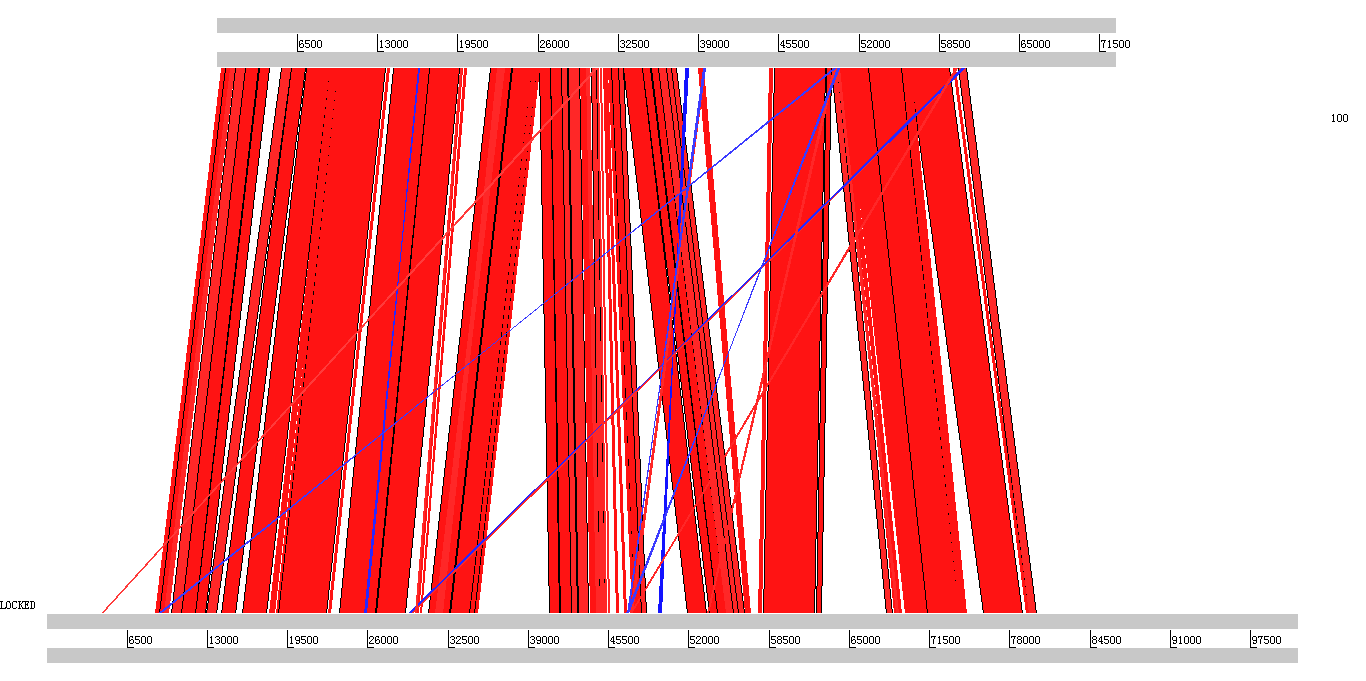  S1-14 | 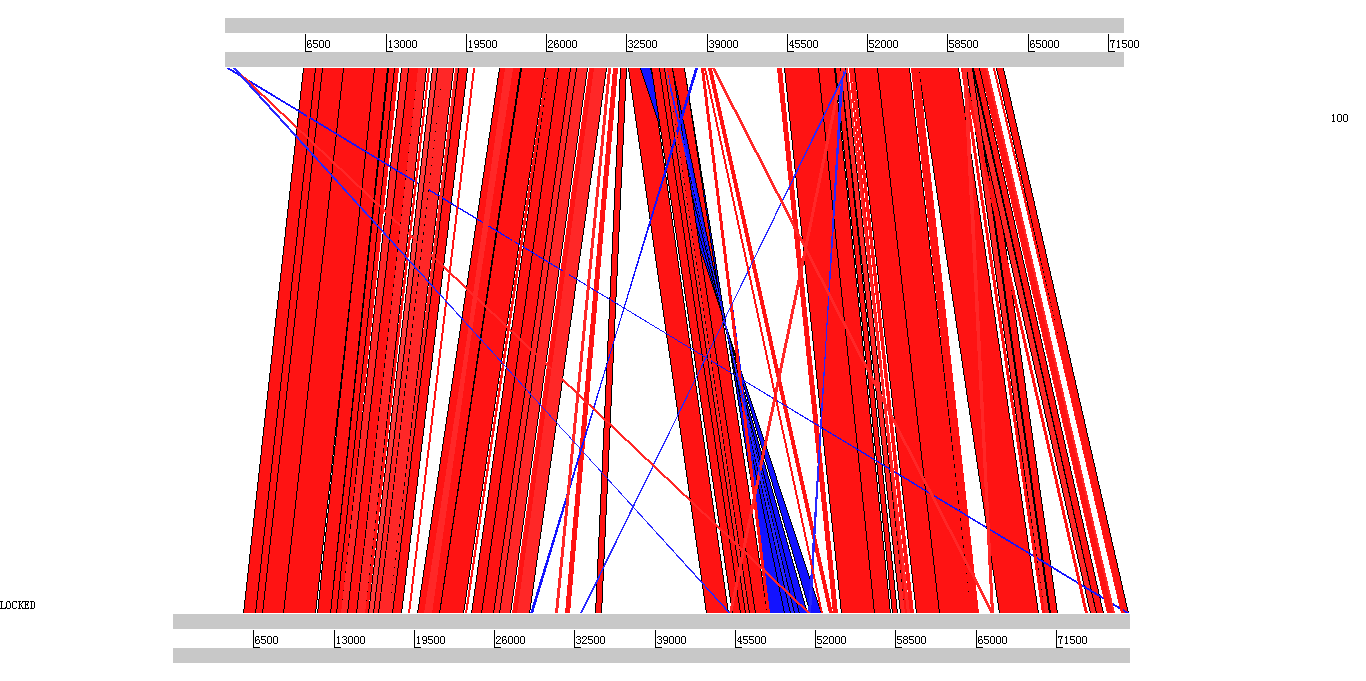  S1-15 |

Additional file 7:**Figure S 1.** Pairwise comparison of BACs sequences from LA-Purple (*S.officinarum*), SES208 (*S. spontaneum*), *Saccarhum* Hybrids( R570)

**Notes:1-1:**Top:SO-57E04(SoI)/Bottom:SO-99P01(SoIII);**1-2:**Top:SO-57E04(SoI)/Bottom: SO-146H19(SoIV);**1-3:**Top:SO-96B11(SoII)/Bottom:SO-99P01(SoIII);**1-4:**Top:SO-96B11(SoII)/Bottom: SO-146H19(SoIV) ;**1-5:** Top: SO-99P01(SoIII) /Bottom: SO-146H19(SoIV); **1-6:**Top: SO-57E04(SoI) /Bottom: SO-96B11(SoII) ; **1-7:**Top: SS-23k06(SsII) /Bottom:SS-75D04(SsI); **1-8:**Top:SS-23k06(SsII)/Bottom:SO-57E04(SoI); **1-9:**Top: SS-23k06(SsII)/Bottom: SO-96B11(SoII);**1-10:**Top:SS-23k06(SsII)/Bottom:SO-99P01(SoIII);**1-11:**Top:SS-23k06(SsII)/Bottom:SO-146H19(SoIV);**1-12:**Top:SS-75D04(SsI)/Bottom:SO-99P01(SoIII); **1-13:**Top:SS-75D04(SsI)/Bottom:SO-57E04(SoI); **1-14:**Top:SS-75D04(SsI)/Bottom: SO-96B11(SoII) ; **1-15:** Top:SS-75D04(SsI) /Bottom: SO-146H19(SoIV).
