# Supplementary material for: Comparative structural analysis of Bru1 region homeologs in Saccharum spontaneum and S. officinarum
Source: BMC Genomics. 2016 Jun 10;17:446. doi: 10.1186/s12864-016-2817-9 (PMC4902974; doi:10.1186/s12864-016-2817-9)
Supplement: Additional file 4: Figure S2. — Mauve visualization of local collinear blocks identified among 14 haplotypes (15BACs) from Saccharum species and sorghum. (DOCX 1909 kb) [file 12864_2016_2817_MOESM4_ESM.docx]

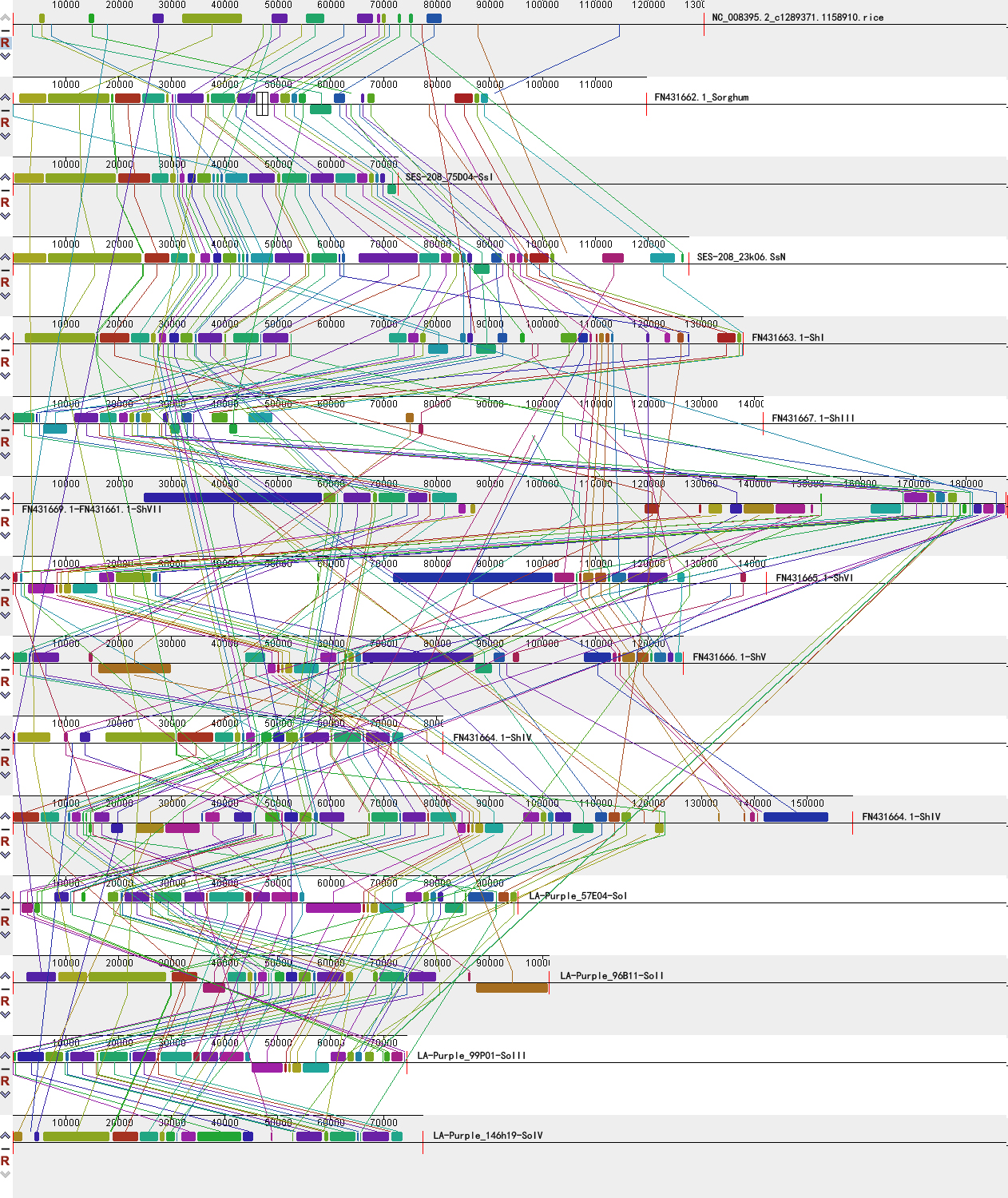


Additional file 8:**Figure S 2.** Mauve visualization of local collinear blocks identified among 14 haplotypes (15BACs) from *Saccharum* species, *Saccharum* hybrid and sorghum.

Homologous local collinear blocks (LCB) were color-coded. LCBs above the center line are in the same orientation as in LA-Purple, whereas LCBs below the center line in the opposite orientation as in LA-Purple.
