# Supplementary material for: Comparative structural analysis of Bru1 region homeologs in Saccharum spontaneum and S. officinarum
Source: BMC Genomics. 2016 Jun 10;17:446. doi: 10.1186/s12864-016-2817-9 (PMC4902974; doi:10.1186/s12864-016-2817-9)
Supplement: Additional file 5: Figure S3. — Gene structure comparison of different haplotype sequences from LA Purple (S. officinarum), AP85-441 (S. spontaneum), and the hybrid cultivar, R570. (DOCX 700 kb) [file 12864_2016_2817_MOESM5_ESM.docx]

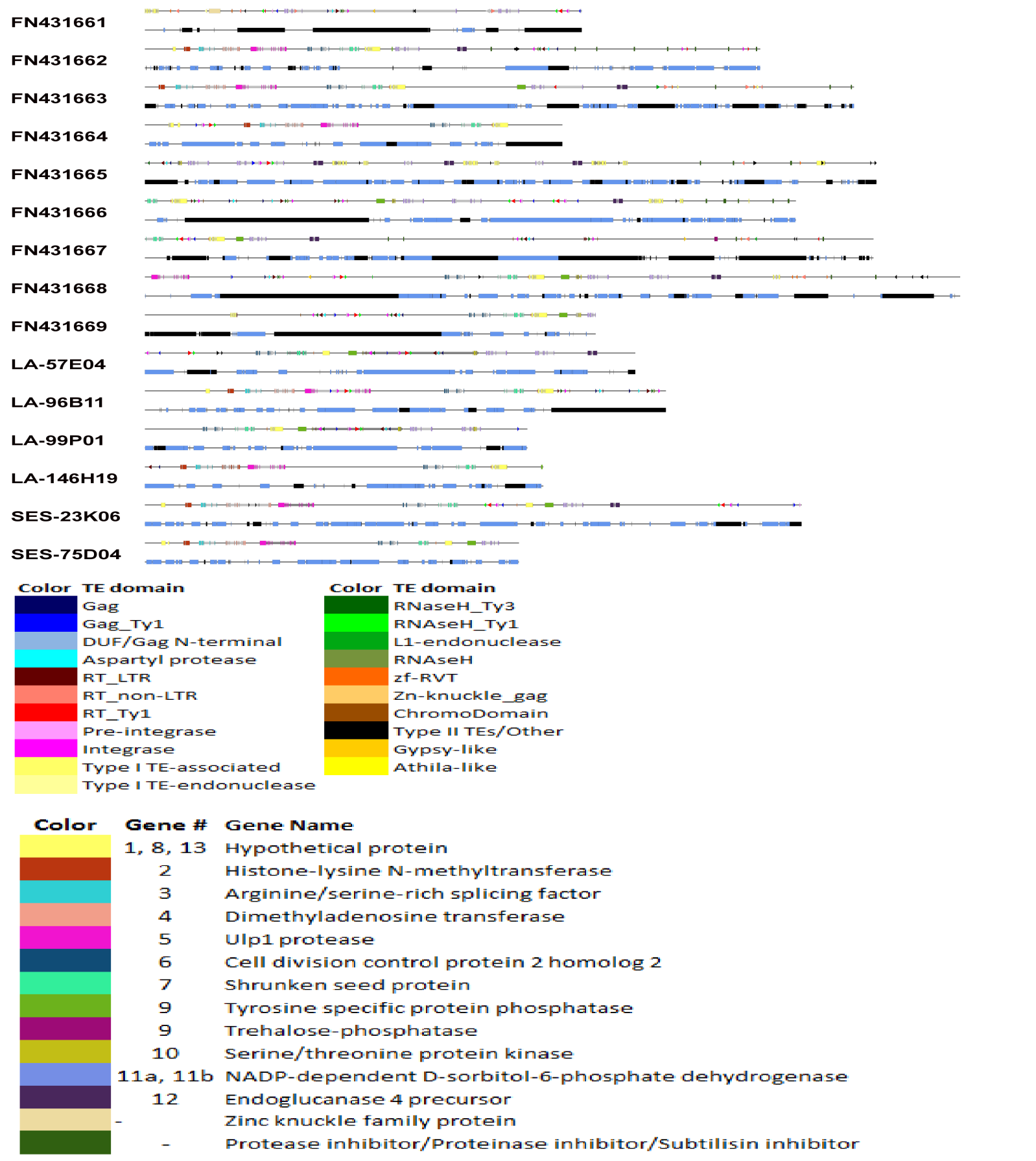

Additional file 9:**Figure S 3.** Genome structures of 14 haplotypes (15BACs) from *Saccharum* species, *Saccharum* hybrid.
