# Supplementary material for: Comparative structural analysis of Bru1 region homeologs in Saccharum spontaneum and S. officinarum
Source: BMC Genomics. 2016 Jun 10;17:446. doi: 10.1186/s12864-016-2817-9 (PMC4902974; doi:10.1186/s12864-016-2817-9)
Supplement: Additional file 6: Figure S4. — The genome structure of haplotypes surrounding the Bru1 locus from LA Purple (S. officinarum), AP85-441 (S. spontaneum), and the hybrid cultivar, R570. (DOCX 95 kb) [file 12864_2016_2817_MOESM6_ESM.docx]

| 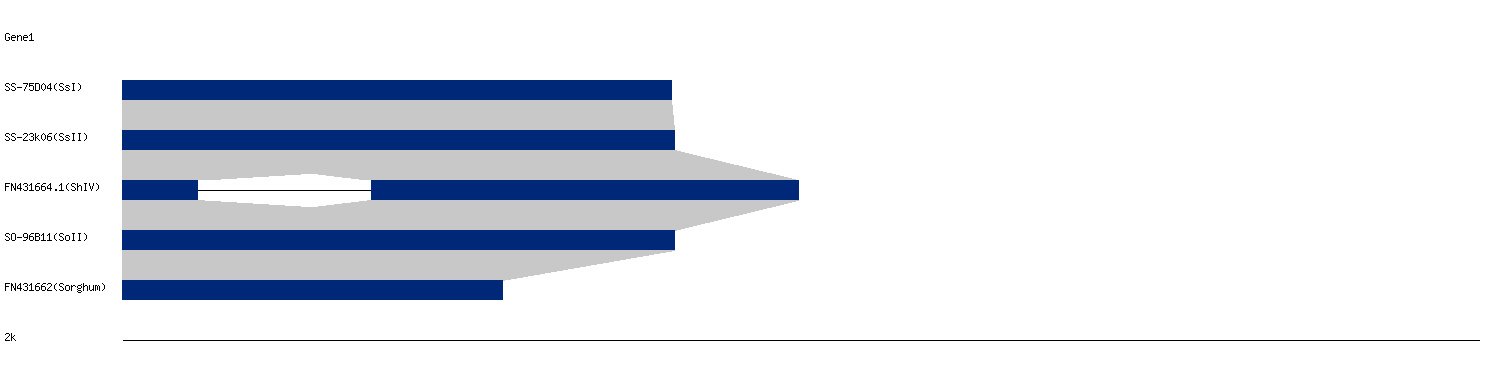 | 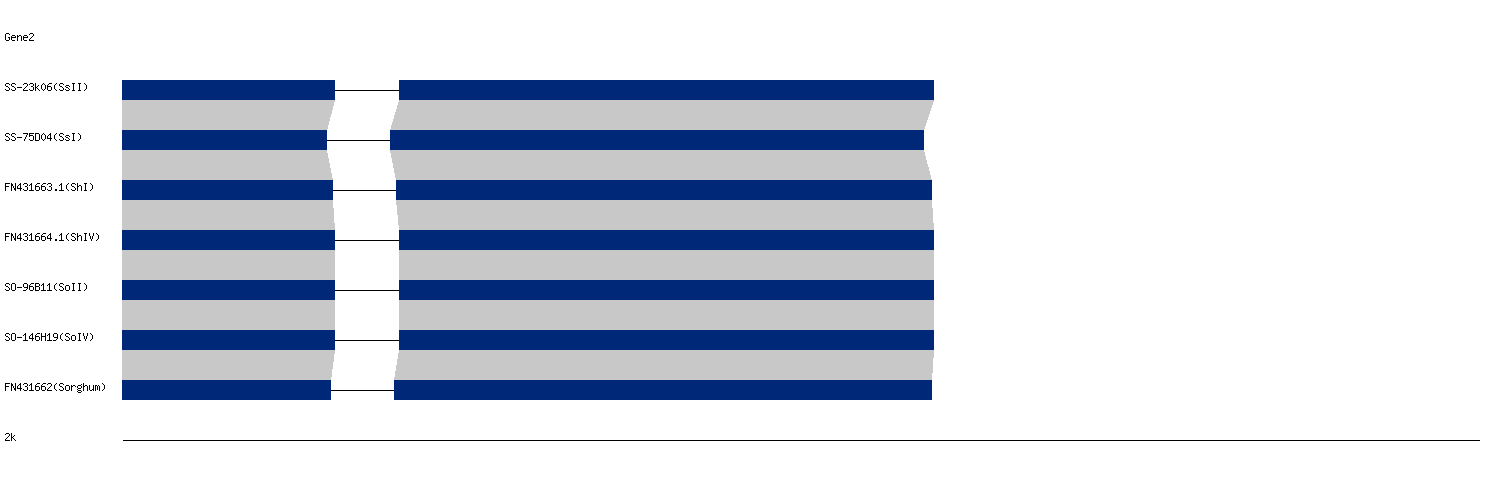 |
| --- | --- |
| Figure S 4.1 | Figure S 4.2 |
| 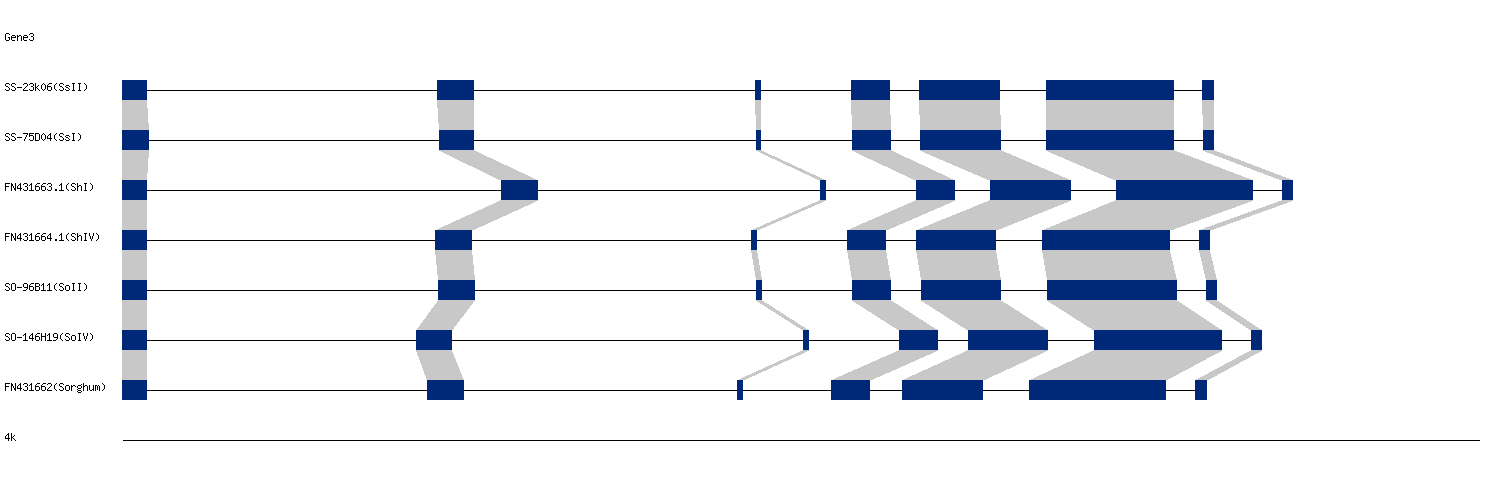 | 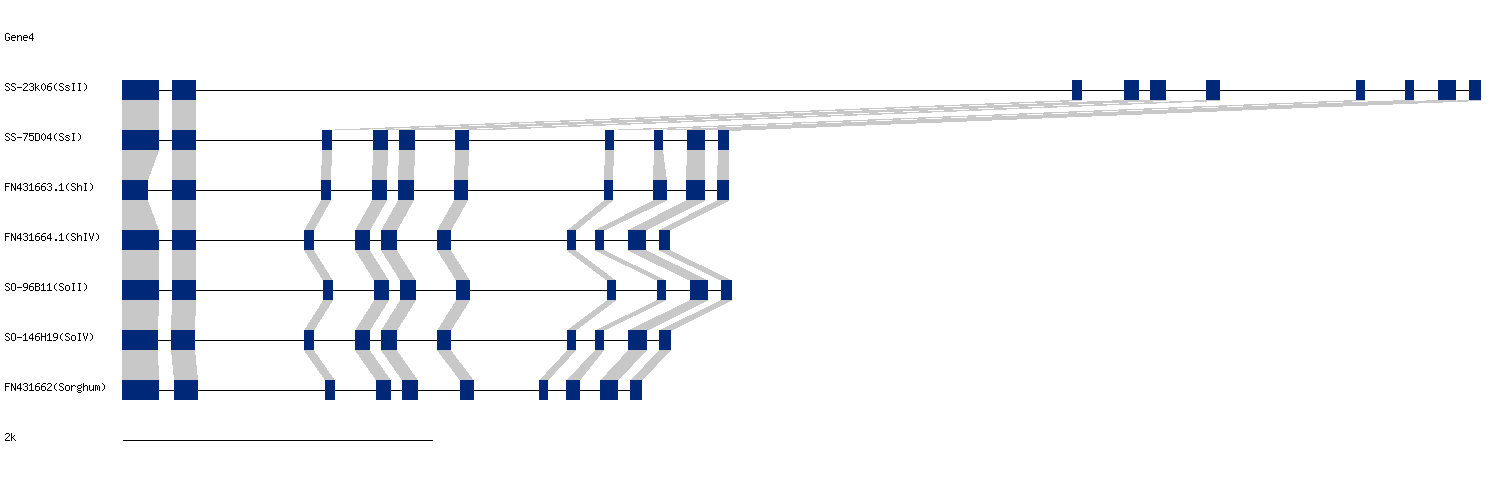 |
| Figure S 4.3 | Figure S 4.4 |
| 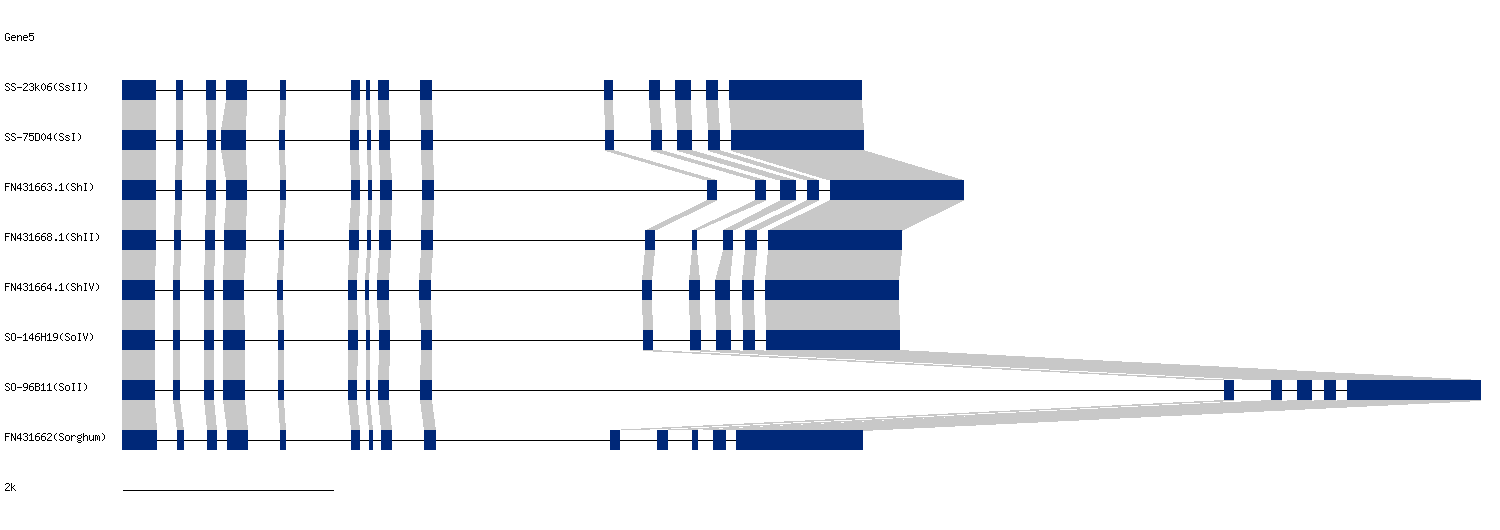 | 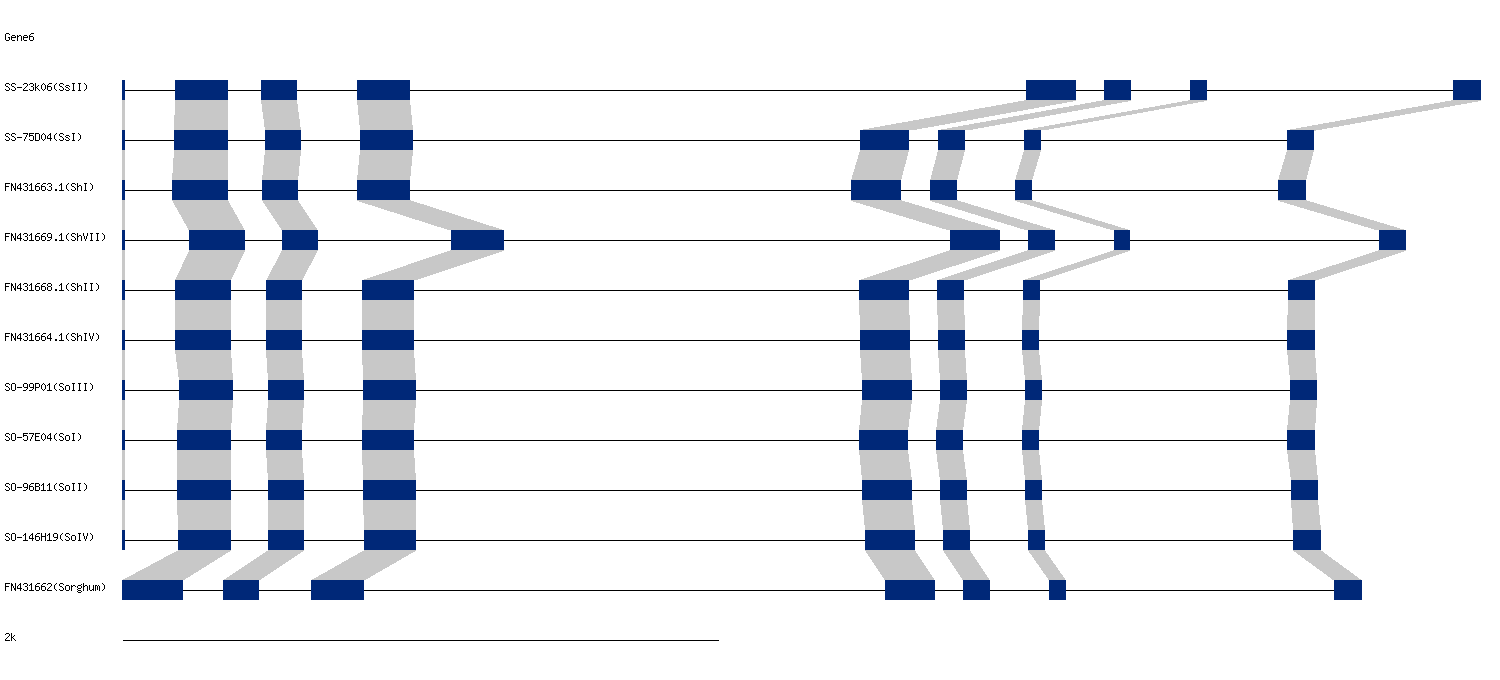 |
| Figure S 4.5 | Figure S 4.6 |

| 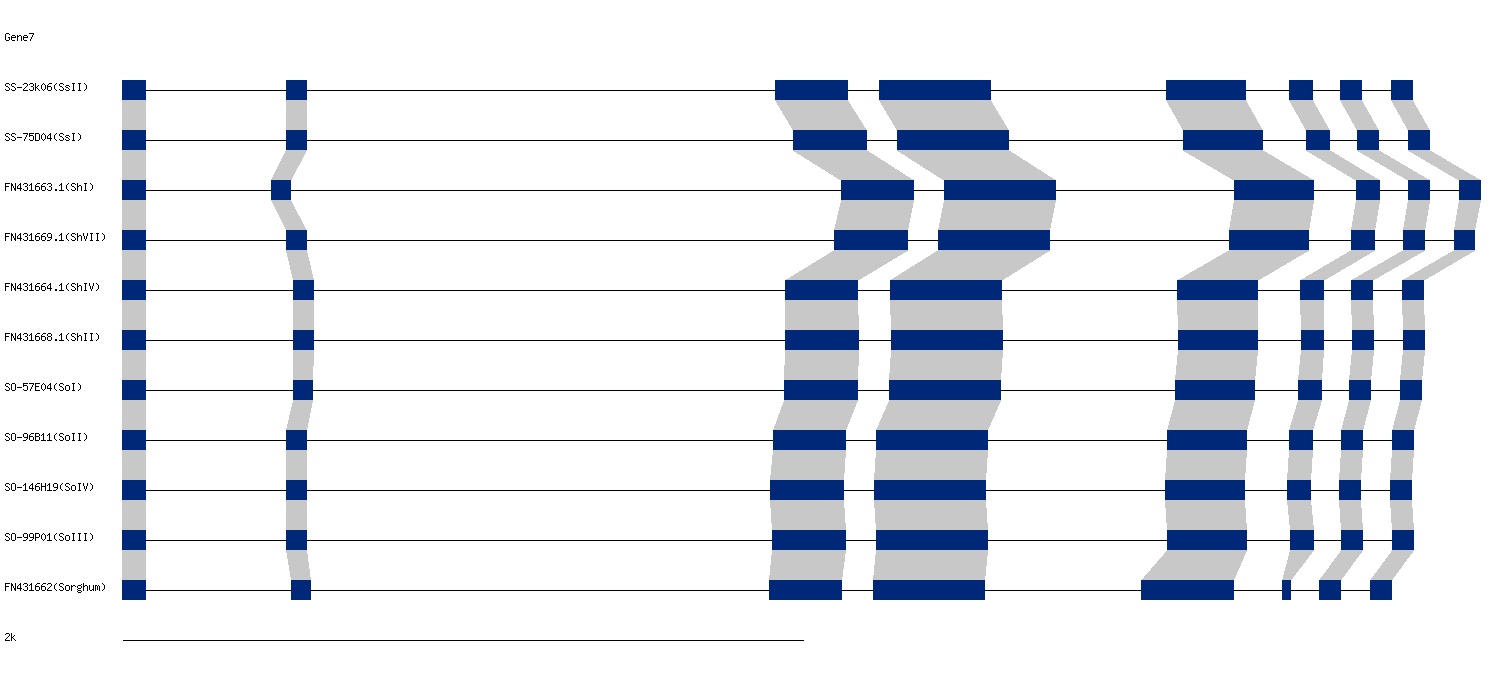 | 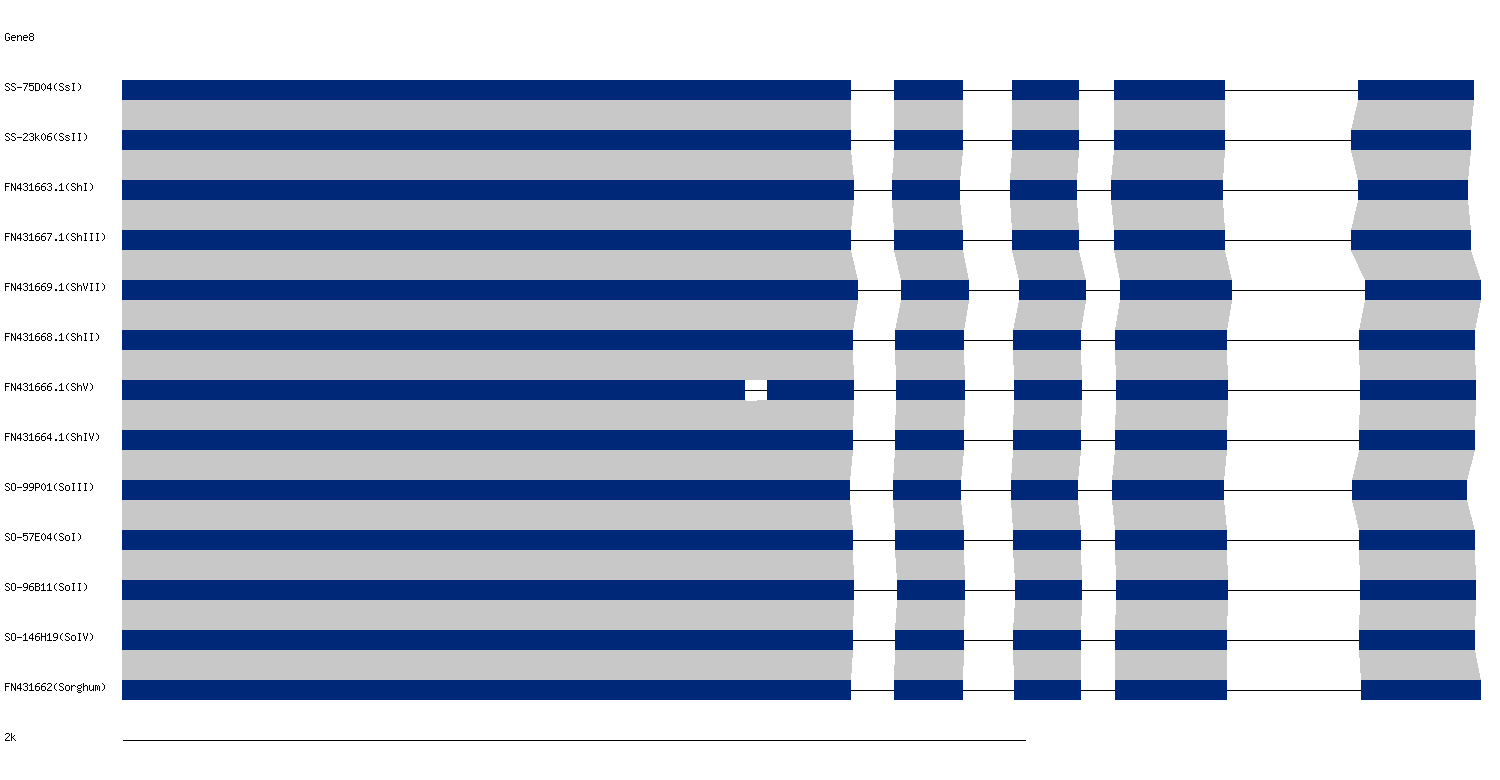 |
| --- | --- |
| Figure S 4.7 | Figure S 4.8 |
| 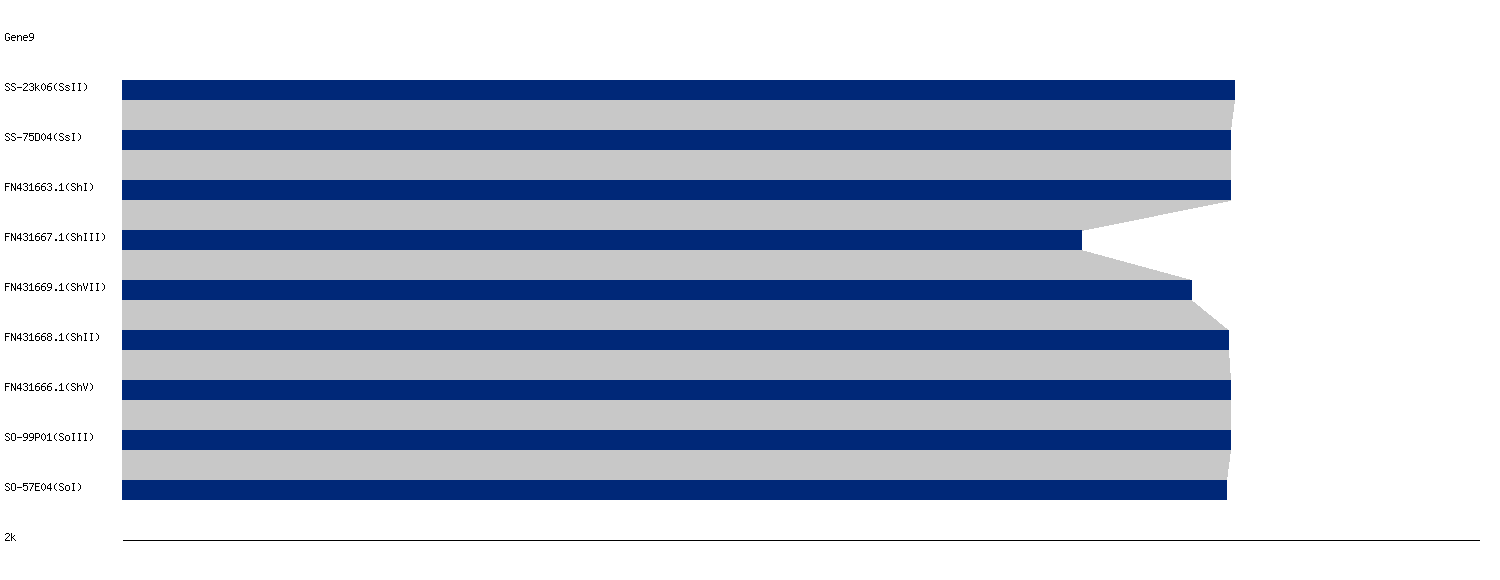 | 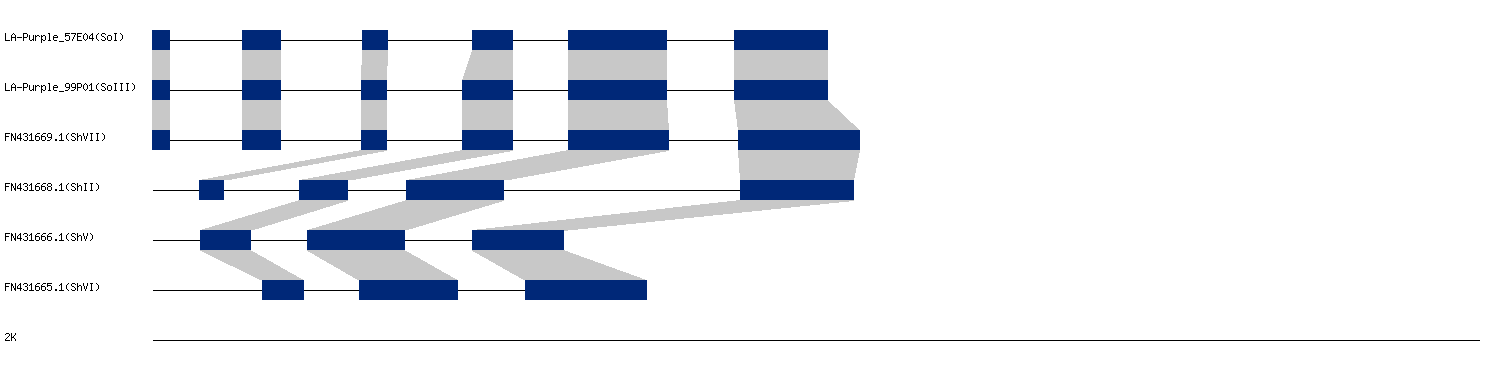 16327  (insertion)  _21273_  _(Insertion)_ |
| Figure S 4.9 | Figure S 4.10 |
| 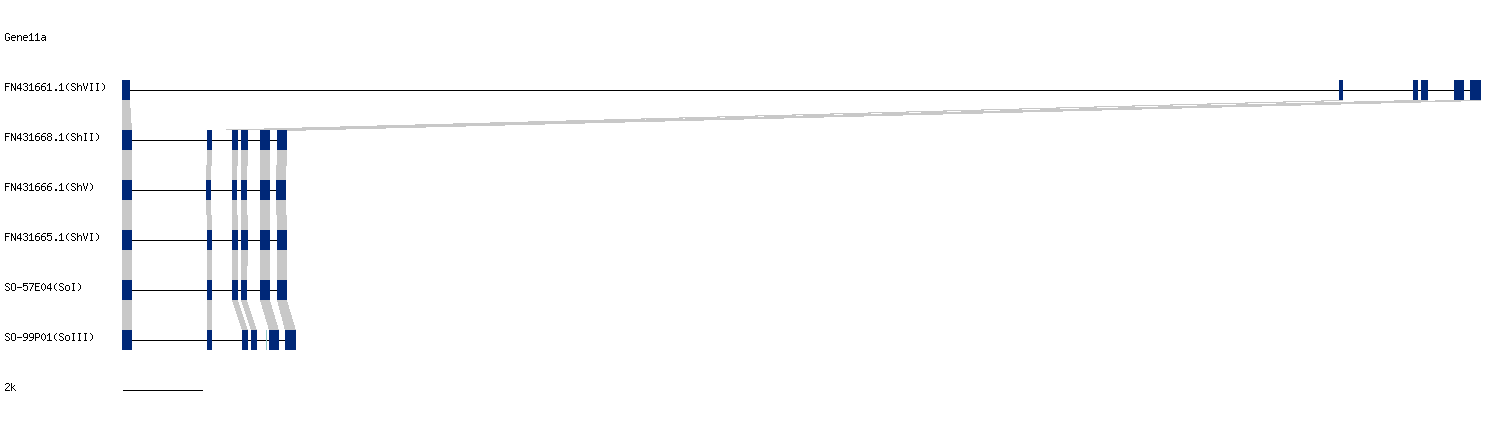  Figure S4.11 | |
| 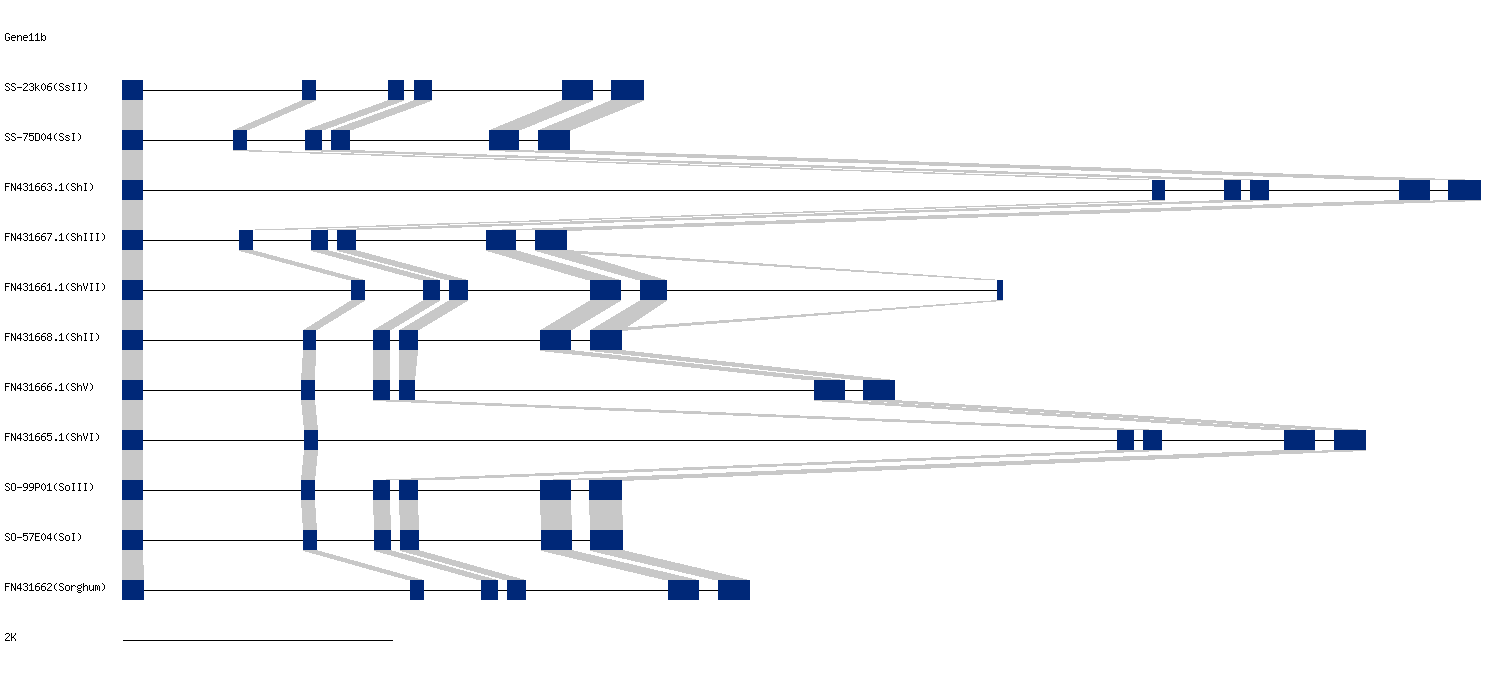Figure S 4.12 | 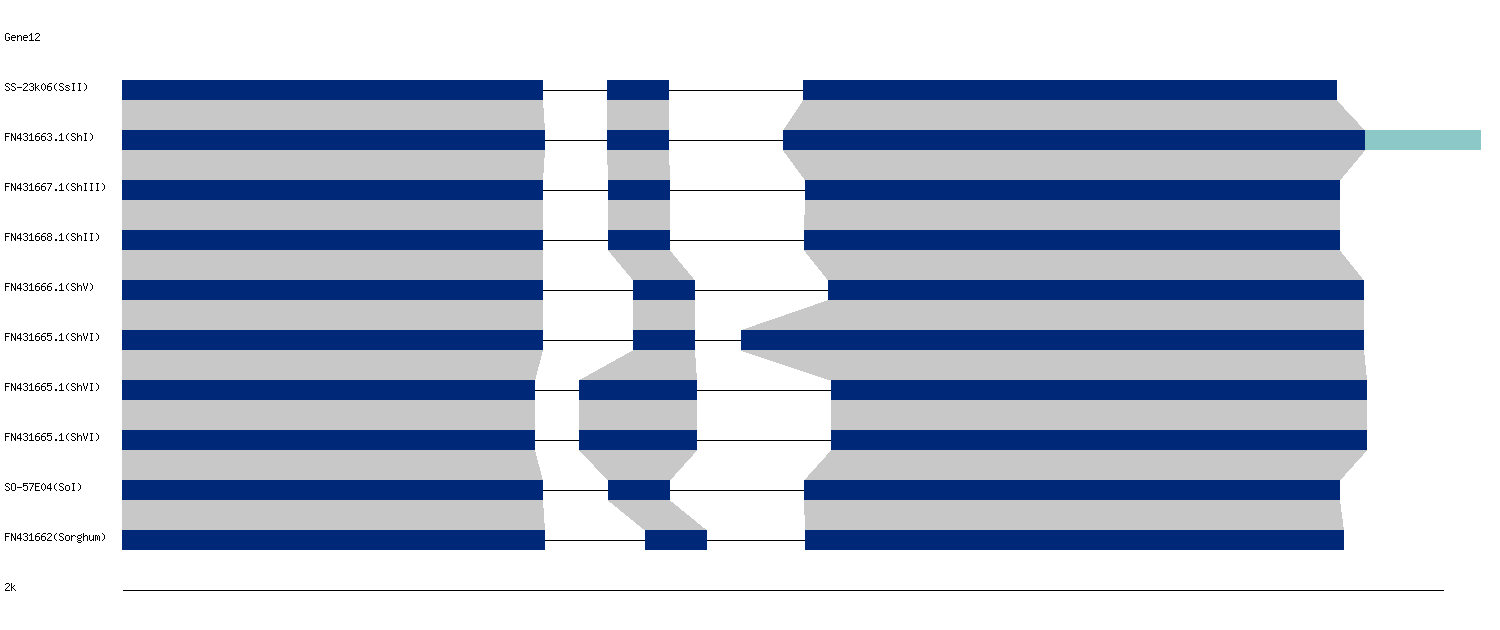  Figure S 4.13 |

Additional file 10:Figure S 4. Comparison of gene structures from different haplotypes among Saccharum speices and Saccharum hybrid.
