# Supplementary material for: Comparative structural analysis of Bru1 region homeologs in Saccharum spontaneum and S. officinarum
Source: BMC Genomics. 2016 Jun 10;17:446. doi: 10.1186/s12864-016-2817-9 (PMC4902974; doi:10.1186/s12864-016-2817-9)
Supplement: Additional file 7: Table S3. — Estimation of synonymous and non-synonymous nucleotide divergence among S. officinarum (LA Purple), S. spontaneum(AP85-441) and hybrid cultivar R570. (DOCX 24 kb) [file 12864_2016_2817_MOESM7_ESM.docx]

| Additional file 3:Table S 3. Estimation of synonymous and nosynymous nucleotide divergence among S.officinarum(LA Purple) ,S.spontaneum(AP85-441) and Saccarhum cultivar R570 | | | | | | | | | | | | | | |
| --- | --- | --- | --- | --- | --- | --- | --- | --- | --- | --- | --- | --- | --- | --- |
|  |  | gene1 | gene2 | gene3 | gene4 | gene5 | gene6 | gene7 | gene8 | gene9 | gene10 | gene11a | gene11b | gene12 |
| *S. spontaneum(AP85-441) - S. spontaneum(AP85-441)* | Gene pairs | 1 | 1 | 1 | 1 | 1 | 1 | 1 | 1 | 1 | - | - | 1 | - |
|  | Average | 1.341 | 0.052 | 0.656 | 0.368 | 0.347 | 0.033 | 0.452 | 0.613 | 0.678 | - | - | 0.186 | - |
| *S. spontaneum(AP85-441) - S. officinarum(LA Purple)* | Gene pairs | 2 | 4 | 3 | 4 | 4 | 8 | 8 | 8 | 2 | - | - | 3 | 1 |
|  | Average | 1.335 | 0.04 | 0.897 | 0.366 | 0.669 | 0.089 | 0.359 | 0.64 | 1.432 | - | - | 0.315 | 0.052 |
| *S. spontaneum(AP85-441) - Saccharum hybrid S.spontaneum* | Gene pairs | - | 2 | 2 | 2 | 2 | 1 | 3 | 4 | 4 | - | - | 4 | 2 |
|  | Average | - | 0.064 | 0.687 | 0.427 | 0.714 | 0.025 | 0.703 | 0.448 | 1.02 | - | - | 0.417 | 0.194 |
| *S. spontaneum(AP85-441) - Saccharum hybrid S.officinarum* | Gene pairs | 2 | 2 | 2 | 2 | 4 | 4 | 4 | 6 | 4 | - | - | 5 | 5 |
|  | Average | 0.549 | 0.033 | 0.557 | 0.412 | 0.737 | 0.071 | 0.304 | 0.711 | 0.921 | - | - | 0.37 | 0.074 |
| *S. spontaneum(AP85-441) - sorghum* | Gene pairs | 2 | 2 | 2 | 2 | 2 | 2 | 2 | 2 | - | - | - | 2 | 1 |
|  | Average | 0.293 | 0.038 | 0.158 | 0.238 | 0.841 | 0.156 | 0.517 | 0.27 | - | - | - | 0.106 | 0.098 |
| *S. officinarum(LA Purple) - S. officinarum(LA Purple)* | Gene pairs | - | - | 1 | 1 | 1 | - | 5 | 6 | - | 1 | 1 | 1 | - |
|  | Average | - | - | 1.37 | 0.417 | 0.79 | - | 0.411 | 0.679 | - | 0.221 | 0.35 | 0.481 | - |
| *S. officinarum(LA Purple) - Saccharum hybrid S.spontaneum* | Gene pairs | - | 2 | 2 | 2 | 2 | 4 | 8 | 8 | 2 | 2 | - | 4 | 2 |
|  | Average | - | 0.088 | 0.82 | 0.356 | 0.49 | 0.042 | 0.467 | 0.615 | 1.018 | 0.777 | - | 0.646 | 0.114 |
| *S. officinarum(LA Purple) - Saccharum hybrid S.officinarum* | Gene pairs | 1 | 2 | 1 | 2 | 4 | - | 11 | 11 | 2 | 5 | 5 | 5 | 5 |
|  | Average | 0.28 | 0.058 | 0.739 | 0.374 | 0.69 | - | 0.312 | 0.769 | 1.852 | 0.559 | 0.249 | 0.611 | 0.126 |
| *S. officinarum(LA Purple) - sorghum* | Gene pairs | 1 | 2 | 2 | 2 | 2 | 4 | 4 | 4 | - | - | - | 2 | 1 |
|  | Average | 0.357 | 0.041 | 0.139 | 0.266 | 0.776 | 0.153 | 0.465 | 0.269 | - | - | - | 0.121 | 0.076 |
| Saccharum hybrid *S.spontaneum* - Saccharum hybrid *S.spontaneum* | Gene pairs | - | - | - | - | - | - | 1 | 1 | 1 | - | - | 1 | 1 |
|  | Average | - | - | - | - | - | - | 0.565 | 0.344 | 0.981 | - | - | 0.422 | 0.24 |
| Saccharum hybrid-*S.spontaneum* - Saccharum hybrid *S.officinarum* | Gene pairs | - | 1 | 1 | 1 | 2 | 2 | 6 | 6 | 4 | - | - | 6 | 10 |
|  | Average | - | 0.061 | 0.549 | 0.434 | 0.593 | 0.044 | 0.34 | 0.671 | 0.777 | - | - | 0.579 | 0.13 |
| Saccharum hybrid *S.spontaneum* - sorghum | Gene pairs | - | 1 | 1 | 1 | 1 | 1 | 2 | 2 | - | - | - | 2 | 2 |
|  | Average | - | 0.039 | 0.178 | 0.227 | 0.707 | 0.138 | 0.553 | 0.286 | - | - | - | 0.108 | 0.134 |
| Saccharum hybrid *S.officinarum* - Saccharum hybrid *S.officinarum* | Gene pairs | - | - | - | - | 1 | - | 2 | 3 | 1 | 3 | 3 | 3 | 10 |
|  | Average | - | - | - | - | 0.992 | - | 0.176 | 0.859 | 1.17 | 0.762 | 0.284 | 0.554 | 0.174 |
| Saccharum hybrid *S.officinarum* - sorghum | Gene pairs | 1 | 1 | 1 | 1 | 2 | 2 | 3 | 3 | - | - | - | 3 | 5 |
|  | Average | 0.312 | 0.041 | 0.103 | 0.312 | 0.781 | 0.136 | 0.433 | 0.281 | - | - |  | 0.128 | 0.106 |
