# Supplementary material for: Comparative structural analysis of Bru1 region homeologs in Saccharum spontaneum and S. officinarum
Source: BMC Genomics. 2016 Jun 10;17:446. doi: 10.1186/s12864-016-2817-9 (PMC4902974; doi:10.1186/s12864-016-2817-9)
Supplement: Additional file 8: Table S4. — The average Ka/Ks ratio of the gene pairs within and between LA Purple (S. officinarum), AP85-441 (S. spontaneum), and the hybrid cultivar, R570. (DOCX 16 kb) [file 12864_2016_2817_MOESM8_ESM.docx]

Additional file 4:Table S 4 The average Ka/Ks ratio for the gene pairs within and between LA Purple (*S.officinarum*), SES208 (*S. spontaneum*), and the hybrid cultivar, R570.

|  |  | *S. spontaneum(SES)* | *S. officinarum(LA Purple)* | Saccharum hybrid-*S.spontaneum* | Saccharum hybrid-*S.officinarum* |
| --- | --- | --- | --- | --- | --- |
| *S. spontaneum*(SES208) | gene pairs | 10 | 47 | 26 | 40 |
|  | average of ka/ks | 0.472^abc^ | 0.473^abc^ | 0.533^ab^ | 0.443^abc^ |
| *S. officinarum*(LA Purple) | gene pairs |  | 16 | 36 | 49 |
|  | average of ka/ks |  | 0.596^a^ | 0.477^abc^ | 0.514^abc^ |
| Saccharum hybrid-*S.spontaneum* | gene pairs |  |  | 5 | 39 |
|  | average of ka/ks |  |  | 0.51^abc^ | 0.417^abc^ |
| Saccharum hybrid-*S.officinarum* | gene pairs |  |  |  | 23 |
|  | average of ka/ks |  |  |  | 0.406^abc^ |
