# Supplementary material for: Comparative structural analysis of Bru1 region homeologs in Saccharum spontaneum and S. officinarum
Source: BMC Genomics. 2016 Jun 10;17:446. doi: 10.1186/s12864-016-2817-9 (PMC4902974; doi:10.1186/s12864-016-2817-9)
Supplement: Additional file 9: Table S5. — Estimated insertion time of full length retrotransposons in Bru1 locus of LA Purple (S. officinarum), AP85-441 (S. spontaneum), and the hybrid cultivar, R570. (DOCX 18 kb) [file 12864_2016_2817_MOESM9_ESM.docx]

Additional file 5:Table S 5: Estimated insertion time of full length retrotransposons in *Bru1* locus of LA Purple (*S.officinarum*), SES208 (*S. spontaneum*), and the hybrid cultivar, R570.

| **TE name** | **Species_CloneName_Accession** | ***k*** | **SD** | **Time (Ma)** | **SD (Ma)** |
| --- | --- | --- | --- | --- | --- |
| 05-05_LTR/copia_S | *S. officinarum_*96B11 | 0.000 | 0.000 | 0.000 | 0.000 |
| 07-08_LTR/copia_AS | *S. spontaneum_*208-23k06 | 0.023 | 0.007 | 0.880 | 0.259 |
| 07-08_LTR/copia_AS | R570_Sh197G04_FN431667.1 | 0.032 | 0.008 | 1.242 | 0.306 |
| 08-09_LTR/gypsy_S | R570_ShCIR12E03_FN431661.1 | 0.001 | 0.001 | 0.021 | 0.020 |
| 10-10_LTR/copia_S | *S. officinarum_*99P01 | 0.026 | 0.004 | 0.992 | 0.149 |
| 10-10_LTR/copia_S | *S. officinarum_*57E04 | 0.033 | 0.004 | 1.278 | 0.148 |
| 10-10_LTR/gypsy_AS | *S. officinarum_*99P01 | 0.011 | 0.006 | 0.442 | 0.228 |
| 10-10_LTR/gypsy_AS | *S. officinarum_*57E04 | 0.026 | 0.009 | 1.004 | 0.351 |
| 12-13_LTR/copia_AS | *S. spontaneum_*208-23k06 | 0.035 | 0.020 | 1.352 | 0.755 |

* The TE name includes three parts separted by underscores: location between genes (e.g., ‘05-05’ means within gene 05, and ‘07-08’ means between genes 07 and 08), type of retroelement (LTR/copia or LTR/gypsy) and orientation (S=Sense, AS=Antisense)

The insertion time was estimated using the conversion rate of 1.3 x 10^-8^ (Ma and Bennetzen, 2004)
