# Supplementary material for: Comparative structural analysis of Bru1 region homeologs in Saccharum spontaneum and S. officinarum
Source: BMC Genomics. 2016 Jun 10;17:446. doi: 10.1186/s12864-016-2817-9 (PMC4902974; doi:10.1186/s12864-016-2817-9)
Supplement: Additional file 10: Table S6. — Summary of the gapless comparisons of pairs of BAC clone sequences from LA Purple (S. officinarum), AP85-441 (S. spontaneum), and the hybrid cultivar, R570. (DOCX 39 kb) [file 12864_2016_2817_MOESM10_ESM.docx]

Additional file 6:Table S.6 Summary of gapless comparison of pair BACs sequences from LA-Purple(*S. officinarum*),SES-208(*S. spontaneum*) and *Saccarhum* Hybrids( R570)
