# Supplementary material for: Comparative structural analysis of Bru1 region homeologs in Saccharum spontaneum and S. officinarum
Source: BMC Genomics. 2016 Jun 10;17:446. doi: 10.1186/s12864-016-2817-9 (PMC4902974; doi:10.1186/s12864-016-2817-9)
Supplement: Additional file 11: Figure S5. — Phylogenetic analysis of 14 haplotypes of genes 11 from Saccharum species, and its homologs from sorghum, Zea mays, and rice. The tree was constructed by the neighbor-joining method implemented in MEGA4 software. The robustness of the tree topology was assessed with 1000 bootstrap replicates. The coding sequences of S6PDH from Zea mays and Malus domestica were used to root the tree. (DOCX 108 kb) [file 12864_2016_2817_MOESM11_ESM.docx]

11a

11b

11a

Additional file 11：**Figure S 5.** Phylogenetic analysis of 14 haplotypes of genes 11 from Saccharum species, and its homologs from sorghum, Zea mays, and rice.

The tree was constructed by the neighbor-joining method implemented in MEGA4 software. The robustness of the tree topology was assessed with 1000 bootstrap replicates. The coding sequences of S6PDH from *Malus domestica* were used to root the tree. The bootstrap values above 60% were showed in the figure.
